# QTL-Seq Analysis for Identification of Resistance Loci to Bacterial Canker in Tomato

**DOI:** 10.3389/fpls.2021.809959

**Published:** 2022-01-26

**Authors:** Alebel Mekuriaw Abebe, Chang-Sik Oh, Hyoung Tae Kim, Giwon Choi, Eunyoung Seo, Inhwa Yeam, Je Min Lee

**Affiliations:** ^1^Department of Horticultural Science, Kyungpook National University, Daegu, South Korea; ^2^Department of Horticultural Biotechnology, College of Life Science, Kyung Hee University, Yongin, South Korea; ^3^Department of Plant and Microbial Biology, University of California, Berkeley, Berkeley, CA, United States; ^4^Department of Horticulture and Breeding, Andong National University, Andong, South Korea

**Keywords:** tomato, bacterial canker, QTL-seq, DNA marker, candidate genes

## Abstract

Bacterial canker caused by *Clavibacter michiganensis* (*Cm*) is one of the most economically important vascular diseases causing unilateral leaf wilting, stem canker, a bird’s-eye lesion on fruit, and whole plant wilting in tomato. There is no commercially available cultivar with bacterial canker resistance, and genomics-assisted breeding can accelerate the development of cultivars with enhanced resistance. *Solanum lycopersicum* “Hawaii 7998” was found to show bacterial canker resistance. A Quantitative trait loci (QTL)-seq was performed to identify the resistance loci using 909 F_2_ individuals derived from a cross between *S. lycopersicum* “E6203” (susceptible) and “Hawaii 7998,” and a genomic region (37.24–41.15 Mb) associated with bacterial canker resistance on chromosome 6 (*Rcm6*) was found. To dissect the *Rcm6* region, 12 markers were developed and several markers were associated with the resistance phenotypes. Among the markers, the Rcm6-9 genotype completely matched with the phenotype in the 47 cultivars. To further validate the *Rcm6* as a resistance locus and the Rcm6-9 efficiency, subsequent analysis using F_2_ and F_3_ progenies was conducted. The progeny individuals with homozygous resistance allele at the Rcm6-9 showed significantly lower disease severity than those possessing homozygous susceptibility alleles. Genomes of five susceptible and two resistant cultivars were analyzed and previously known R-genes were selected to find candidate genes for *Rcm6*. Nucleotide-binding leucine-rich repeat, receptor-like kinase, and receptor-like protein were identified to have putative functional mutations and show differential expression upon the *Cm* infection. The DNA markers and candidate genes will facilitate marker-assisted breeding and provide genetic insight of bacterial canker resistance in tomato.

## Introduction

Bacterial canker of tomato (*Solanum lycopersicum*) is a destructive disease caused by a Gram-positive actinomycete *Clavibacter michiganensis* (*Cm*) and was first detected in 1909, United States (Smith, 1910; Nandi et al., 2018). The infected plants may show unilateral wilting, marginal leaf necrosis, stem canker, stunted plant growth, and small dark spots surrounded by a whitish margin on fruits (Sen et al., 2015; Peritore-Galve et al., 2020). The damage due to bacterial canker depends on plant growth stage, location, cultivar, weather condition, and inoculum concentration (Forster and Echandi, 1973). The yield losses caused by the bacterial canker range from 46 to 93% and result in a significant decrease in fruit weight during the highest disease incidence under field condition (Chang et al., 1992; Poysa, 1993).

The *Cm* is a seed-borne pathogen infecting the vascular tissue and fruits in tomato. *Cm* can survive for extended period in infested seeds and leftover debris or a short period in the soil (Tsiantos, 1987; Sen et al., 2015). The *Cm* enters into the host through natural openings (stomata and hydathodes) or wounds on the surface of leaves, roots, and stems, which later moves into the xylem tissue to proliferate and multiply (Bae et al., 2015; Nandi et al., 2018). The *Cm* multiplies extensively within the xylem lumen and fills it with bacterial aggregates (Chalupowicz et al., 2012; Peritore-Galve et al., 2020). Moreover, it secretes extracellular cell wall degrading enzymes, such as cellulase, polygalacturonase, pectate lyase, and xylanase to degrade xylem vessels and the adjacent parenchyma cells leading to induction of disease symptom (Gartemann et al., 2008; Hwang et al., 2019). Although chemical, biological, and cultural practices might reduce bacterial canker infestation in the field (Hausbeck et al., 2000), breeding for resistant cultivars is the sustainable approach in tomato (Crino et al., 1995; Stüwe and von Tiedemann, 2013). To date, no tomato cultivar of bacterial canker resistance is commercially available and cultivated tomatoes are vulnerable to a bacterial canker disease outbreak (Sen et al., 2015). A wide range of tomato germplasm collections was evaluated to find new resistance sources to bacterial canker (Poysa, 1993; Sotirova et al., 1994; Sandbrink et al., 1995; Francis et al., 2001; Sen et al., 2013).

Genetic analysis of bacterial canker resistance has been studied on a few accessions of *S. habrochaites*, *S. arcanum*, and *S. pimpinellifolium* (Sandbrink et al., 1995; van Heusden et al., 1999; Kabelka et al., 2002; Coaker and Francis, 2004; Sen, 2014). Quantitative trait loci (QTL) mapping using a backcross population from *S. arcanum* “LA2157,” identified five resistance loci on chromosomes 1, 6, 7, 8, and 10. A QTL analysis using the F_2_ population of *S. arcanum* “LA2157” identified resistance loci on chromosomes 5, 7, and 9 showing an additive interaction (van Heusden et al., 1999). A QTL analysis using a BC_2_S_5_ population in *S. habrochaites* “LA407” mapped resistance loci on chromosomes 2 (*Rcm 2.0*) and 5 (*Rcm 5.1*). The *Rcm 2.0* and *Rcm 5.1* explained 25.7–34.0 and 25.8–27.9% of the phenotypic variation, respectively (Coaker and Francis, 2004). In *S. pimpinellifolium* “GI1554,” five QTLs on chromosomes 1, 2, 7, 8, and 12 were identified using recombinant inbred lines (RIL; Sen, 2014). In *S. lycopersicum*, “Hawaii 7998,” “IRAT L3,” and “Okitsu Sozai I-20” were reported as resistant sources (Steekelenburg, 1985; Gardner et al., 1990). However, any QTL analysis of bacterial canker resistance using *S. lycopersicum* was not conducted.

Understanding the host-pathogen interaction is essential to explain the molecular resistance/susceptibility mechanism. Defense response to pathogen attack mediated by different resistance (R) genes can follow various signaling and immune activation mechanisms. Recently, nine molecular mechanisms followed by R-genes to activate disease resistance have been suggested. These include cell-surface recognition mechanisms (direct or indirect), intracellular perception mechanisms (direct, indirect, integrated, and executor), and loss of susceptibility mechanisms (active, passive, and reprogram; Kourelis and van der Hoorn, 2018). The mechanism of *Cm* resistance in tomato is not well-understood as no resistance gene is identified yet. The proteome analysis of both *Cm* and tomato during infection highlighted potential proteins involved in disease development and basal defense response, which can be targets to further understand *Cm*-tomato interaction (Savidor et al., 2012). The *Cm* proteome analysis during the infection revealed the induction of proteins involved in signal perception and transduction. This is followed by the production of proteases and pectate lyases, which might target host proteins. In turn, tomato initiates signal transduction and activates basal defense response to *Cm* infection (Coaker et al., 2004; Savidor et al., 2012). Microarray analysis of tomato during *Cm* infection identified differential expressed genes involved in defense response and biosynthesis of phytohormones (Balaji et al., 2008). A comparative transcriptome analysis was performed on *Cm*–resistant and–susceptible tomato lines, and defense-responsive genes were differentially expressed (Basim et al., 2021). The microscopic analysis of vascular stem sections in *S. lycopersicum* “IRAT L3,” showed a larger and increased number of tyloses than in the *Cm* susceptible cultivars (Stüwe and von Tiedemann, 2013). This feature may help to limit the spread of the pathogen within the vascular system of the resistant cultivar.

A bulked segregant analysis (BSA) is a rapid and cost effective method for detecting QTL in populations with extreme phenotypic variation (Michelmore et al., 1991). The QTL-seq combines whole-genome resequencing and conventional BSA to rapidly detect genomic regions linked to the trait of interest (Takagi et al., 2013). It has been applied to detect loci associated with many traits in various crops (Zou et al., 2016).

To identify genomic regions associated to bacterial canker resistance in *S. lycopersicum* “Hawaii 7998,” QTL-seq approach was employed, and the DNA markers were developed and validated. A candidate QTL on chromosome 6 was identified and designated as *Rcm6*. Insertion/Deletion (InDel) markers developed in the *Rcm6* can be used for marker assisted selection in tomato breeding against bacterial canker. Furthermore, the identified candidate genes and underlying mutations will provide better insights for understanding bacterial canker resistance in tomato.

## Materials and Methods

### Plant Materials

Tomatoes were grown in glasshouse of Kyungpook National University at an average temperature of 25–28°C and 16/8 h light/dark cycles. An F_2_ population derived from a cross between “E6203” (susceptible) and “Hawaii 7998” (resistant) was used for the QTL-seq analysis. The F_2_ population in experiment-I (*n* = 250), II (*n* = 340), and III (*n* = 319) along with 10 plants of each parent were inoculated. A set of 47 tomato cultivars (listed in Table 1), F_2_ population, and F_3_ progenies were used for marker validation.

**Table 1:** Genotyping of various tomato cultivars with InDel markers in the *Rcm6* interval.

Cultivar	Disease severity^a^	Phenotype	Marker genotype^b^											
			Rcm6-1	Rcm6-2	Rcm6-3	Rcm6-4	Rcm6-5	Rcm6-6	Rcm6-7	Rcm6-8	Rcm6-9	Rcm6-10	Rcm6-11	Rcm6-12
Super High Power	1.1 ± 0.13a	Resistant	R	R	R	R	R	R	R	R	R	R	R	R
Hawaii 7998	1.2 ± 0.13a	Resistant	R	R	R	R	R	R	R	R	R	R	R	R
Hawaii 7996	1.3 ± 0.17a	Resistant	R	R	R	R	R	R	R	R	R	R	R	R
B-Blocking	1.4 ± 0.16a	Resistant	R	R	R	R	R	R	R	R	R	R	R	R
High Power	1.4 ± 0.16a	Resistant	H	H	R	R	R	R	R	R	R	R	R	R
IRAT L3	1.4 ± 0.22a	Resistant	R	R	R	R	R	R	R	R	R	R	R	R
BWR-20	1.5 ± 0.40a	Resistant	R	R	R	R	R	R	R	R	R	R	S	S
Shincheonggang	1.6 ± 0.16a	Resistant	R	R	R	R	R	R	R	R	R	R	R	R
Spider	2.0 ± 0.39ab	Resistant	R	R	R	R	R	R	R	R	R	H	H	R
SVTX6258	2.0 ± 0.47ab	Resistant	R	R	H	H	H	S	R	H	R	R	R	R
Fighting	2.0 ± 0.53ab	Resistant	R	R	R	R	R	R	R	R	R	R	R	R
10-BA-3-33	3.0 ± 0.15bc	Susceptible	S	S	S	S	S	S	S	S	S	S	S	S
Florida 7481	3.2 ± 0.92cd	Susceptible	S	S	S	S	S	S	S	S	S	S	S	S
10-BA-4-24	3.3 ± 0.15c-e	Susceptible	S	S	S	S	S	S	S	S	S	S	S	S
UC-134	3.3 ± 0.68c-e	Susceptible	S	S	S	S	S	S	S	S	S	S	S	S
Miniheuksu	3.6 ± 0.52c-f	Susceptible	S	S	S	S	R	S	R	S	S	S	S	S
C-5	3.6 ± 0.68c-f	Susceptible	S	S	S	S	S	S	S	S	S	S	S	S
Santa Cruz B	3.8 ± 0.58c-g	Susceptible	S	S	S	S	S	S	S	S	S	S	S	S
Anahu	4.0 ± 0.55c-g	Susceptible	S	S	S	S	S	S	S	S	S	S	S	S
Angela	4.0 ± 0.55c-g	Susceptible	S	S	S	S	S	S	S	S	S	S	S	S
AVT-2	4.0 ± 0.51c-g	Susceptible	S	S	S	S	S	S	S	S	S	S	S	S
Motelle	4.2 ± 0.80d-g	Susceptible	H	S	S	S	H	S	S	S	S	H	R	R
New Yorker	4.2 ± 0.49d-g	Susceptible	S	S	S	S	S	S	S	S	S	S	S	S
Yellow Peach	4.2 ± 0.58d-g	Susceptible	S	S	S	S	S	S	S	S	S	S	S	S
Dotaerang Red	4.3 ± 0.39e-g	Susceptible	S	S	S	S	S	S	R	S	S	S	S	S
M82	4.4 ± 0.24e-g	Susceptible	S	S	S	S	S	S	S	S	S	S	S	S
Black Plum	4.4 ± 0.60e-g	Susceptible	S	S	S	S	S	S	S	S	S	S	S	S
Gold Nugget	4.4 ± 0.60e-g	Susceptible	S	S	S	S	S	S	R	S	S	S	S	S
Indigo Rose	4.4 ± 0.26e-g	Susceptible	S	S	S	S	S	S	S	S	S	S	S	S
Heinz 1350	4.5 ± 0.34fg	Susceptible	S	S	S	S	S	S	S	S	S	S	S	S
Dafnis	4.6 ± 0.40fg	Susceptible	S	S	S	S	S	S	S	S	S	S	H	R
Black Cherry	4.6 ± 0.31fg	Susceptible	R	S	S	S	S	S	S	S	S	S	S	S
A-1	4.8 ± 0.20g	Susceptible	S	S	S	S	S	S	H	S	S	S	S	S
Rowpac	4.8 ± 0.20g	Susceptible	S	S	S	S	S	S	S	S	S	S	S	S
Super Dotaerang	4.8 ± 0.20g	Susceptible	S	S	S	S	S	S	S	S	S	S	S	S
VF36	4.9 ± 0.10g	Susceptible	S	S	S	S	S	S	S	S	S	S	S	S
KN009	5.0 ± 0.00g	Susceptible	R	S	S	S	H	R	H	S	S	R	R	R
E6203	5.0 ± 0.00g	Susceptible	S	S	S	S	S	S	S	S	S	S	S	S
Heinz 1706	5.0 ± 0.00g	Susceptible	S	S	S	S	S	S	S	S	S	S	S	S
Ailsa Craig	5.0 ± 0.00g	Susceptible	S	S	S	S	S	S	S	S	S	S	S	S
Money maker	5.0 ± 0.00g	Susceptible	S	S	S	S	S	S	S	S	S	S	R	R
Purple 1	5.0 ± 0.00g	Susceptible	S	S	S	S	S	S	S	S	S	S	S	S
Purple 2	5.0 ± 0.00g	Susceptible	S	S	S	S	S	S	S	S	S	R	R	R
YT2359	5.0 ± 0.00g	Susceptible	S	S	S	S	R	S	R	S	S	R	R	R
CP-2	5.0 ± 0.00g	Susceptible	S	S	S	S	S	S	S	S	S	S	S	S
Florida 8516	5.0 ± 0.00g	Susceptible	S	S	S	S	S	S	S	S	S	S	S	S
Pinkle	5.0 ± 0.00g	Susceptible	R	S	S	S	R	R	R	S	S	R	R	R

a Mean disease severity followed by different letters is significantly different (*p* < 0.05) according to Duncan’s multiple range test.

b Marker genotype. R, homozygous Hawaii 7998 genotype; H, Heterozygous genotype; and S, homozygous E6203 genotype.

### Pathogen Inoculation and Disease Evaluation

The pathogen *Cm* strain LMG 7333 was cultured on King’s B (KB) medium (proteose peptone 20 g, dipotassium hydrogen phosphate 1.5 g, 1 M magnesium sulfate 6 ml, 50% glycerol 16 ml, and agar 15 g per 1 L of distilled water). The culture was incubated for 48 h at 26°C. The bacterial culture was washed with 10 mM MgCl_2_ to make the inoculum suspension and the concentration was adjusted to approximately 10^8^ CFU/ml (OD_600_ = 0.4) using a smart spec plus spectrophotometer (Bio-Rad Laboratories, Inc. Singapore). For inoculation, seeds were germinated in Petri-dish (90 mm) and transferred to 50 cell-trays. One-month-old tomato seedlings (5–6 leaf stage) were inoculated with the leaf clipping method (Hwang et al., 2020), and disease symptom was evaluated at 5 weeks post-inoculation. Sterilized scissors were infected by dipping in bacterial suspension and four leaflets of each plant were cut diagonally with the infected scissors. The inoculated plants were kept in a growth chamber (temperature = 26°C; Relative Humidity = 60%; light/dark = 16/8 h.). Disease severity was rated based on 0–5 disease scale where: 0 = no visible symptom; 1 = 0–25% leaves wilting; 2 = 26–50% leaves wilting; 3 = 51–75% leaves wilting; 4 ≥ 76% leaves wilting; and 5 = whole plant wilting and dead (Mohd Nadzir et al., 2019).

### Genomic DNA Extraction

The genomic DNA (gDNA) used in this study was isolated from young leaf tissues using a modified cetyltrimethyl ammonium bromide (CTAB) method (Murray and Thompson, 1980). The quality and quantity of gDNA were checked using NanoDrop 2000/UV-Vis spectrophotometer (ThermoFisher Scientific, Waltham, MA, United States).

### Construction of Bacterial Canker-Resistant and Susceptible Bulks and Whole-Genome Resequencing

Young leaves were collected from “E6203,” “Hawaii 7998,” and F_2_ individuals before inoculation. For the construction of bulks, 36 susceptible (disease severity score: 5; S-bulk1) and 36 resistant (disease severity score: 0 and 1; R-bulk1) F_2_ individuals were selected in experiment-I. In experiment-II, 34 F_2_ individuals were selected for each susceptible (S-bulk2) and resistant (R-bulk2) bulks. The gDNAs of resistant and susceptible bulks were prepared from equal amounts of gDNA from each F_2_ individual in the pool. A library of ~350 bp insert size was constructed at Macrogen (Macrogen Inc., Daejeon, Korea) using TruSeq DNA PCR-Free kit (Illumina, Inc.; San Diego, CA, United States); according to the manufacturer’s instructions to obtain 151 bp paired-end reads. The whole-genome resequencing of four bulks (S-bulk1, R-bulk1, S-bulk2, and R-bulk2), and “E6203” was carried out using an Illumina HiSeq 4000 instrument (Illumina, Inc.; San Diego, CA, United States) with Hiseq Sequencing kits. The resequencing of “Hawaii 7998” was previously conducted (Kim et al., 2018).

### Sequencing Data Analysis and Identification of Candidate Genomic Region for Bacterial Canker

Raw data were trimmed using trimmomatic-0.36 (Bolger et al., 2014). The reads from “E6203” and “Hawaii 7998” were mapped to a reference sequence (SL3.0) of *S lycopersicum* cv. Heinz 1706 (CM001064.3–CM001075.3; Tomato Genome Consortium, 2012) using BWA-MEM (Li and Durbin, 2009) to generate reference sequences of “E6203” and “Hawaii 7998.” Then, R-bulk and S-bulk sequences from both experiments were mapped to the reference sequence of “E6203” and “Hawaii 7998” using BWA-MEM. The variant callings were performed using HaplotypeCaller in Genome analysis toolkit (GATK; McKenna et al., 2010). All vcf files were read by vcfR (Knaus and Grünwald, 2017) in R 3.4.3 (R Core Team, 2013), and SNPs were filtered out by low coverage depth (<10).

The SNP-index was defined as the ratio between the number of reads of an alternative SNP and the total number of reads corresponding to the SNP. The SNP-index is equal to 1 when nucleotides of all bulk reads are different from nucleotides of reference at the same position and is equal to 0 when nucleotides of all bulk reads are identical to a nucleotide of the reference at the same position (Takagi et al., 2013). Therefore, it was expected that causal regions for canker resistance would be close to 1 in S-bulk and would be less than 0.5 in R-bulk when the “Hawaii 7998” sequence was used as a reference. In contrast, when the “E6203” sequence was used as a reference, it was expected that causal regions for canker resistance would be close to 1 in R-bulks and less than 0.5 in S-bulks. ∆(SNP-index) was calculated according to the following formula: Δ(SNP-index) = R-bulk SNP-index – S-bulk SNP-index, when “E6203” was used as a reference and; Δ(SNP-index) = S-bulk SNP-index – R-bulk SNP-index, when “Hawaii 7998” was used as a reference. The average SNP-index was calculated for a 100 kb window interval with 10 kb increments. Based on the null hypothesis, a 95% CI was used to determine candidate genomic region for *Cm* resistance.

### Development of InDel Markers in the Candidate Region

Sequence variation in the *Rcm6* between “E6203” and “Hawaii 7998” was extracted from the whole-genome resequencing data, and InDel markers dissecting the region were developed. PCR was carried out in a total volume of 25 μl containing 100 ng of DNA using e-*Taq* DNA Polymerase according to the manufacturer’s instructions (SolGent, Daejeon, Korea). Amplification was carried out using Bio-Rad T100™ thermal cycler (Bio-Rad Laboratories, Inc., Singapore) with the following conditions: initial cycle denaturation at 95°C for 3 min, 34 cycles at 95°C for 30 s, annealing at 50.1–57.5°C (varies for different primers) for 30 s, extension at 72°C for 1 min, and the last cycle at 72°C for 5 min. The PCR amplicons were visualized by electrophoresis under 0.5% TBE buffer in 3% agarose gel stained with ethidium bromide. Forty-seven tomato cultivars, F_2_ population, and F_3_ progenies were genotyped to validate the association of the putative QTL with bacterial canker resistance.

### Identification of Candidate Genes for Bacterial Canker Resistance

Candidate genes were mined within the *Rcm6* interval according to the tomato reference genome annotation (ITAG3.2; https://solgenomics.net/), based on their putative function in disease resistance. Genes encoding proteins of gene families that previously identified as R-genes (Zhang et al., 2019; Deng et al., 2020) were selected and subjected to sequence variation analysis. Candidate genes were compared for sequence variation between susceptible (E6203, Heinz 1706, Moneymaker, Ailsa Craig, and Black Cherry) and resistant (Hawaii 7998 and Hawaii 7,996) cultivars. The sequence variant information of Ailsa Craig, Moneymaker, and Blackcherry were obtained from Tomato Genomic Variations database (http://psd.uohyd.ac.in/tgv; Gupta et al., 2020). “Hawaii 7996” and “Hawaii 7998” were previously sequenced (Kim et al., 2018), and “E6203” was resequenced in this study. Genes harboring the putative amino acid sequence variation between the susceptible and resistant cultivars were selected as candidates for bacterial canker resistance. Protein domains of putative resistance genes were predicted using the Pfam database^1^ and SMART.^2^

### Candidate Gene Sequencing

To validate the sequence variation of candidate genes based on the publicaly available genome data, nine candidate genes harboring putative functional mutations were selected and sequenced in five susceptible and two resistant cultivars. The gDNA was amplified using *e*-*Taq* DNA polymerase (SolGent Co., Ltd., Daejeon, Korea) and primers (Supplementary Table 1) according to the manufacturer’s instructions. The PCR products were purified using MG PCR/Gel Combo kit (MGmed, Daejeon, Korea) and sequenced (SolGent Co., Ltd., Daejeon, Korea).

### Gene Expression Analysis

To analyze the expression of the candidate genes, leaf tissues were collected from 4-week-old plants of “E6203” and “Hawaii 7998” at 0-day post-inoculation (dpi; mock-inoculation), 2 dpi, and 4 dpi (*n* = 3/each), and immediately frozen in liquid nitrogen. Total RNA was isolated from 100 mg of frozen and ground leaf tissues using TRIzol reagent (Thermo Fisher Scientific, United States) and quantified using a Nanodrop 2000 Spectrophotometer (Thermo Fisher Scientific, United States). The first strand cDNA was synthesized from total RNA using DiaStar™ RT Kit (SolGent Co., Ltd., Daejeon, Korea). Quantitative RT-PCR was conducted using the *Power* SYBR® Green PCR master mix (Applied Biosystems™, United States) and gene-specific primers (Supplementary Table 1) in a StepOne™ Real-Time PCR System (Applied Biosystems™, United States) according to the manufacturer’s instructions with two technical replicates per sample. The tomato *GAPDH* (*glyceraldehyde-3-phosphate dehydrogenase*; *Solyc05g014470.3.1*) was used to normalize the expression levels (Takishita et al., 2018).

## Results

### Disease Evaluation of Parental Lines and F_2_ Population

**Figure 1:**
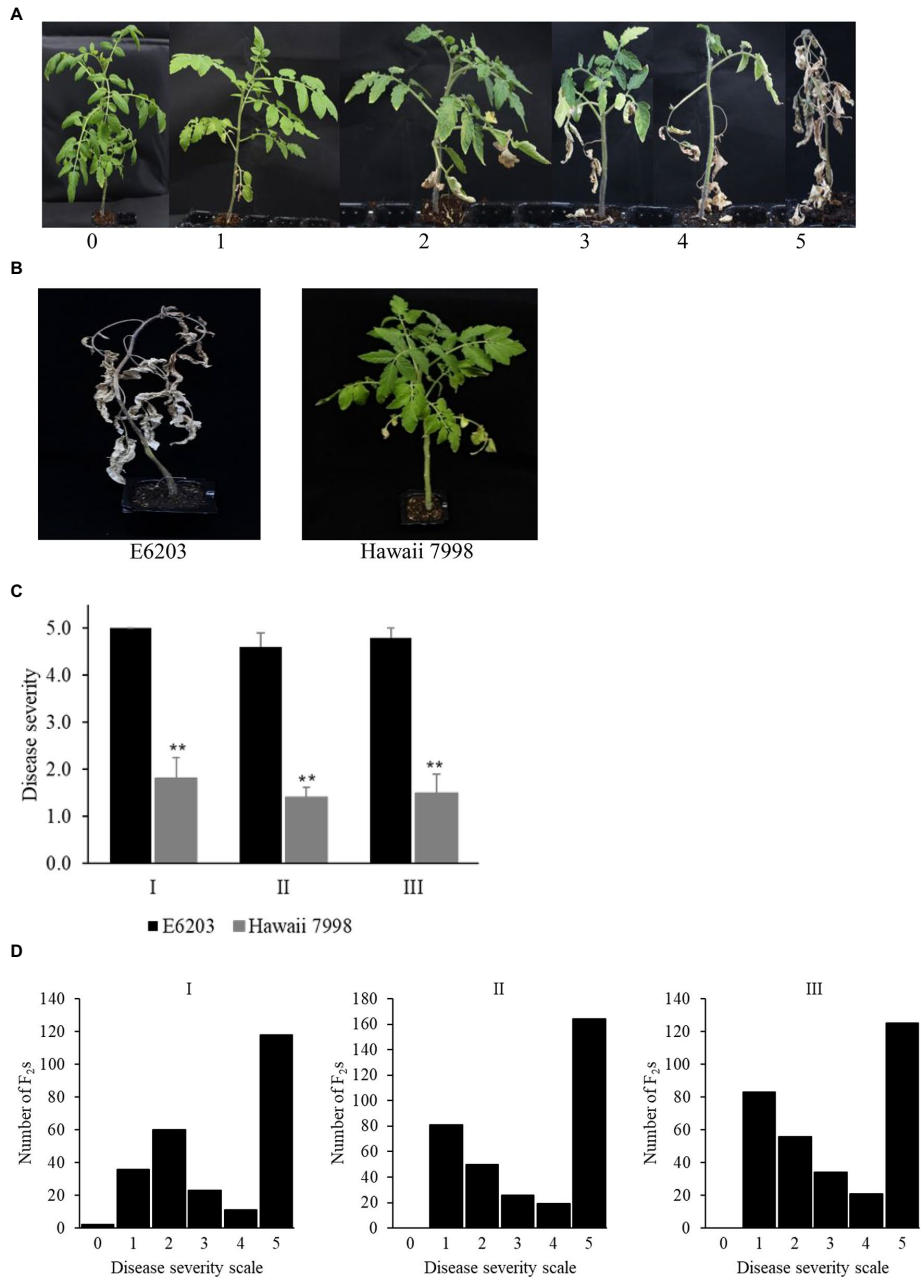
Evaluation of parental lines and F_2_ population for bacterial canker resistance. **(A)** Disease severity scale (0–5) at 5 weeks post-inoculation against *Cm*. **(B)** Phenotypes of susceptible “E6203” and resistant “Hawaii 7998” at 5 weeks post-inoculation against *Cm*. **(C)** Disease severity of “E6203” and “Hawaii 7998” against *Cm* inoculation in three independent experiments. The asterisks indicate a significant difference as determined by Student’s t-test at *p* < 0.01. **(D)** Frequency distribution of disease severity scales of the F_2_ population in experiment-I, II, and III.

The F_2_ population derived from a cross between “E6203” (susceptible) and “Hawaii 7998” (resistant), and the parents were inoculated with *Cm* strain LMG 7333. The disease severity was evaluated in three independent experiments based on the disease severity scale (0–5; Figure 1A). The susceptible cultivar “E6203” showed 4.6 ± 0.31–5.0 ± 0.00 of disease severity score while the resistant cultivar “Hawaii 7998” showed 1.2 ± 0.00–1.8 ± 0.44 of disease severity score (Figures 1B,C). In total, 909 F_2_ plants were evaluated for bacterial canker resistance in three independent experiments. The F_2_ plants exhibited continuous frequency distribution of the disease severity (Figure 1D). The disease score distribution in the F_2_ population showed that susceptible F_2_ individuals were prevalent in all the three experiments.

### Whole-Genome Resequencing of Parents and Bacterial Canker-Resistant and Susceptible F_2_ Bulks

The whole genome of parental lines and the F_2_ bulks from experiments I and II were resequenced using Illumina HiSeq4000. A total of 70,055,398 and 213,327,144 reads were generated with an average depth of approximately 11.75 and 35.79X for “E6203” and “Hawaii 7998” (Kim et al., 2018), respectively. Similarly, the bulk sequencing resulted in 70,609,818 (S-bulk1), 68,848,412 (R-bulk1), 251,792,964 (S-bulk2), and 235,851,694 (R-bulk2) reads with an average depth of 11.84, 11.55, 42.24, and 39.57X, respectively. The Q30 values ranged between 90.01 and 94.00% indicating the high quality of the produced sequences (Supplementary Table 2).

### QTL-Seq Analysis and Candidate Genomic Region for Bacterial Canker Resistance

**Figure 2:**
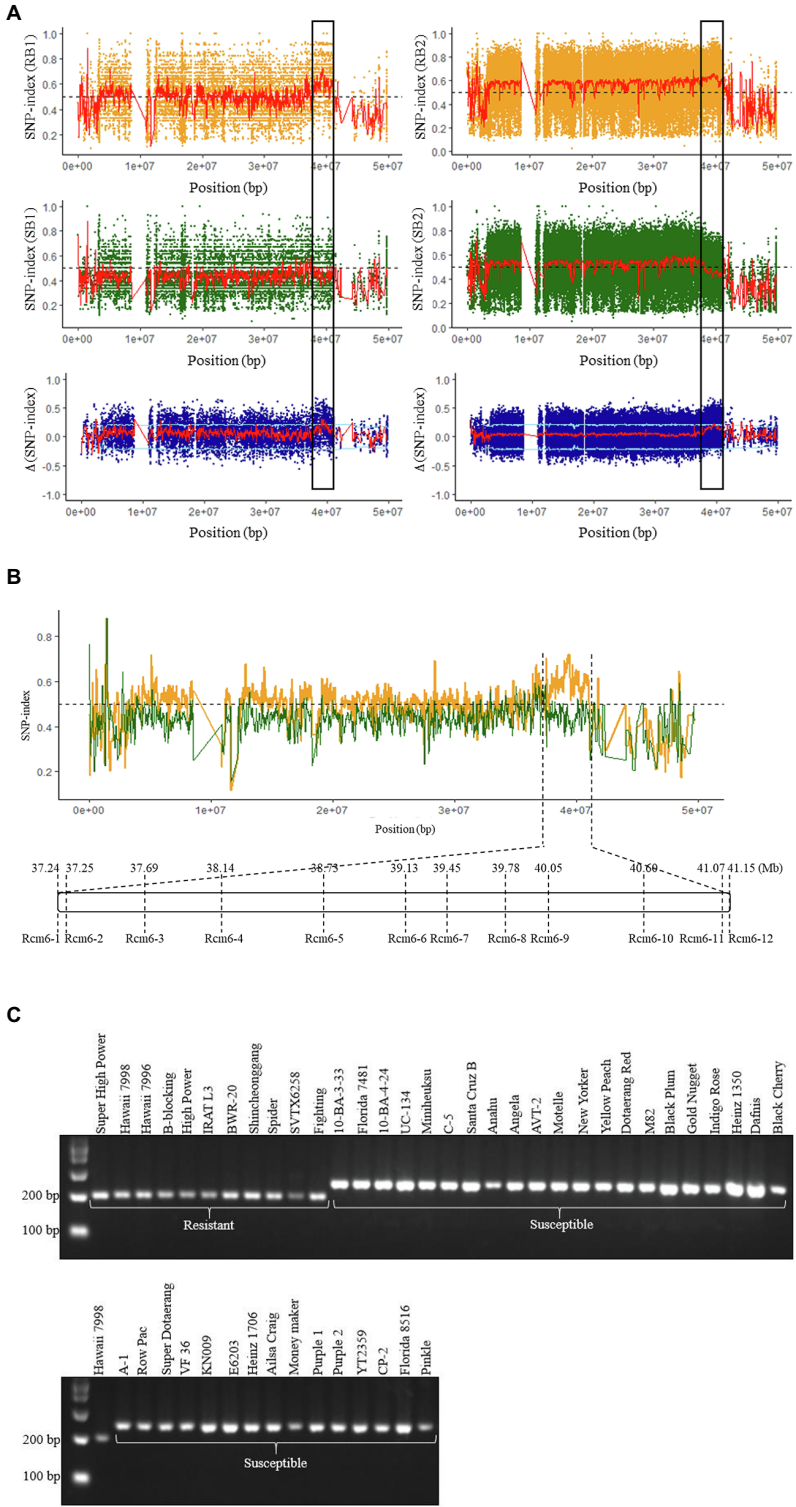
Candidate genomic region for bacterial canker resistance and schematic location of InDel markers in the *Rcm6*. **(A)** SNP-index plots for chromosome 6 of resistant (orange), susceptible (dark green) bulks, and Δ(SNP-index; dark blue) with the “E6203” as a reference in experiment-I (left) and II (right). Red lines indicate the sliding window average of 100 kb interval with 10 kb increments for SNP-index. Δ(SNP-index) was obtained by subtracting the susceptible bulk SNP-index from the resistant bulk SNP-index. Light blue line indicates the statistical CI at significance level (*p* < 0.05). Black boxes indicate candidate genomic region for bacterial canker resistance. **(B)** Schematic location of InDel markers in *Rcm6*. Average SNP-index plots for chromosome 6 of the resistant (orange) and susceptible (dark green) bulks with “E6203” as a reference in experiment-I. **(C)** Genotyping of Rcm6-9 in 47 tomato cultivars. Eleven resistant and 36 susceptible cultivars were discriminated by Rcm6-9 marker.

SNP-index was calculated for each SNP in S-bulk and R-bulk in comparison to the reference genome assemblies of both parents. The average SNP-indices of S-bulk and R-bulk as well as the Δ(SNP-index), were calculated for 100 kb window intervals with a 10 kb increment to detect candidate genomic regions. The SNP-index plots were generated for all 12 chromosomes in experiments I and II using genomes of “E6203” and “Hawaii 7998” as references (Supplementary Figures S1–S4). The genomic regions showing peaks or valleys in the Δ(SNP-index) plot or highly opposite trends of SNP-index for S-bulk and R-bulk to SNP-index value of 0.5 are likely to contain QTL associated with the trait (Takagi et al., 2013; Wu et al., 2019). SNP-indices of S-bulk and R-bulk appear as mirror images with respect to the line of SNP-index = 0.5 on chromosome 6 between 37.24–41.15 Mb. S-bulk has the genomic segment of “E6203” type, where R-bulk has the genomic segment of “Hawaii 7998” type in this interval. This interval was commonly identified in both experiments I and II. The average Δ(SNP-index) of the window showed consistent peaks at this region, which is identified as a candidate QTL (*Rcm6*) of bacterial canker resistance (Figure 2A; Supplementary Figure S5).

### Marker Development and Validation

**Figure 3:**
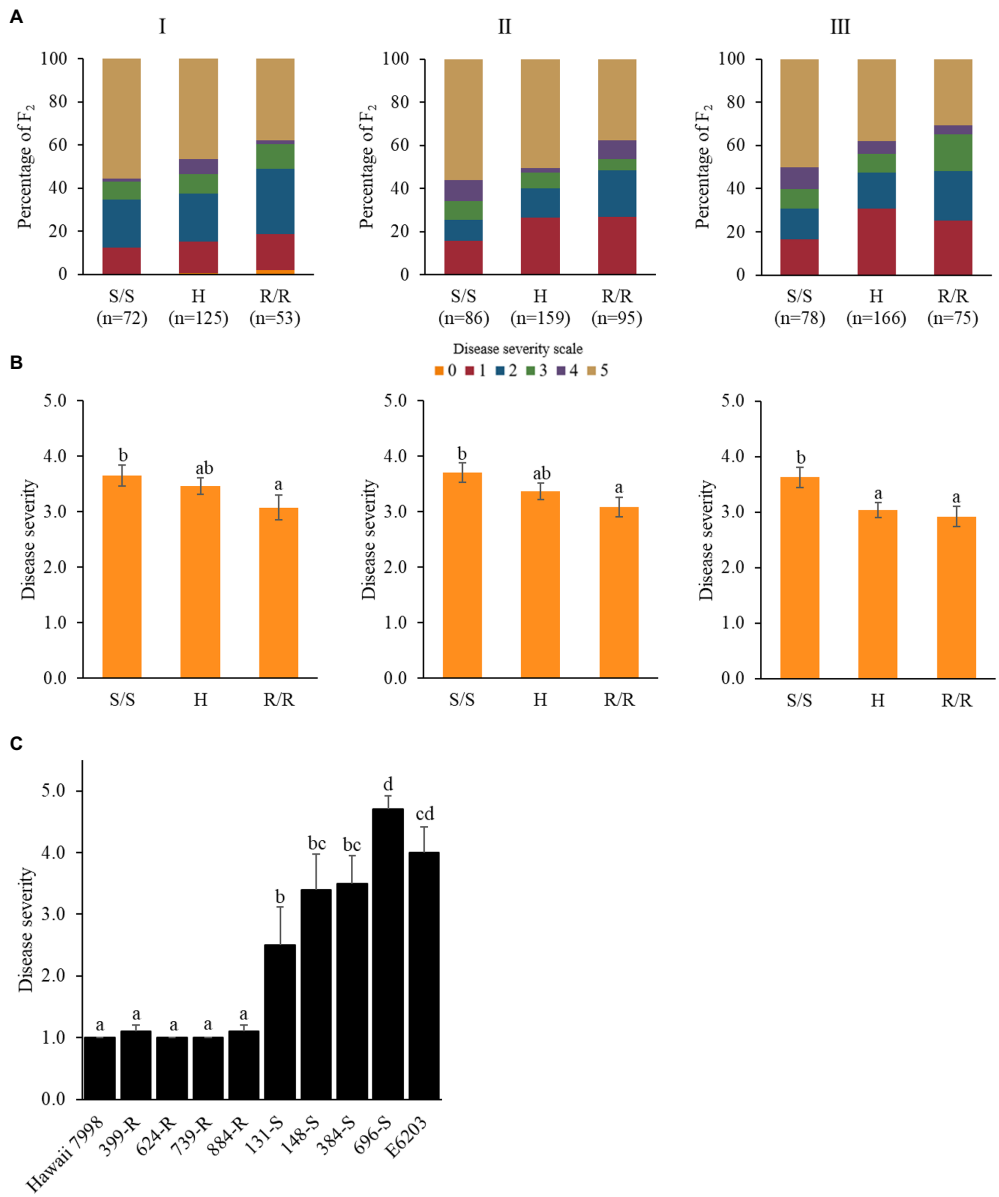
Performance of Rcm6-9 in the F_2_ population derived from “E6203” and “Hawaii 7998” in three experiments. **(A)** Percentage of F_2_ with each disease scale within the genotypes. **(B)** Mean values with different letters on the bars are significantly different (*p* < 0.05) according to Duncan’s multiple range test. *S* represents the susceptible “E6203” allele, *H* represents the heterozygote, and *R* represents the resistant “Hawaii 7998” allele. **(C)** Disease severity of the F_3_ progenies harboring homozygous “Hawaii 7998” alleles (R) and homozygous “E6203” alleles (S) of Rcm6-9 at 5 weeks post-inoculation against Cm. Mean values with different letters on the bars are significantly different (*p* < 0.05) according to Duncan’s multiple range test.

To validate the identified genomic region, *Rcm6* was dissected by 12 InDel markers (Figure 2B; Supplementary Table 3). Forty-seven tomato cultivars were evaluated for bacterial canker resistance including previously reported cultivars such as resistant “IRAT L-3” (Stüwe and von Tiedemann, 2013) and susceptible “Moneymaker” (Mohd Nadzir et al., 2019). For the qualitative resistance screening, cultivars were classified as resistant (disease severity score ≤ 2.0) and susceptible (disease severity score ≥ 3.0; Bartkiewicz et al., 2018; Abebe et al., 2020). Accordingly, 11 cultivars were grouped as resistant and 36 as susceptible (Table 1). All 12 markers were genotyped to 47 tomato cultivars. The genotype in two regions of *Rcm6* (Rcm6-2–Rcm6-4 and Rcm6-8–Rcm6-9) was consistent with the respective cultivars’ phenotypes. The Rcm6-2, Rcm6-3, Rcm6-4, and Rcm6-8 genotypes showed one heterozygous genotype and completely matched the phenotype of 46 cultivars (Table 1). The Rcm6-9 genotype was completely matched with the phenotype in the 47 tomato cultivars (Figure 2C) with 100% of true positive and true negative rates (Supplementary Table 4). The Rcm6-9 performance analysis in the F_2_ population indicated that disease severity was significantly lower in progenies harboring homozygous “Hawaii 7998” allele than progenies harboring “E6203” allele in all three experiments (Figures 3A,B). Similarly, Rcm6-1, Rcm6-5, and Rcm6-12 were evaluated in the F_2_ population. “Hawaii 7998” type alleles of Rcm6-1 showed significantly lower disease severity in experiment I and II. In case of Rcm6-5 and Rcm6-12, disease severity showed significant difference only in experiment I and II, respectively (Supplementary Figure S6). Furthermore, to develop a marker-assisted selection system for bacterial canker resistance, the F_3_ progenies were used for Rcm6-9 validation. Four homozygous resistant and four susceptible F_3_ progenies to Rcm6-9 were evaluated for bacterial canker resistance. F_3_ progenies harboring homozygous “Hawaii 7998” alleles of Rcm6-9 showed significantly lower disease severity than F_3_ progenies harboring homozygous “E6203” alleles of Rcm6-9 (Figure 3C).

### Identification of Candidate Genes for *Rcm6*

*Rcm6* was highly associated with bacterial canker resistance based on the marker analysis in the germplasms, F_2_ population, and F_3_ progenies (Table 1; Figure 3; Supplementary Figure S6). *Rcm6* (Rcm6-1–Rcm6-12) region contains 463 genes according to the tomato reference genome annotation (ITAG 3.2). Potential candidate genes were scanned in the interval based on their putative function. Genes encoding nucleotide-binding domain and leucine-rich repeat (NLR), receptor-like kinases (RLK), and receptor-like proteins (RLP), which account majority of identified R-genes (Kourelis and van der Hoorn, 2018), were found in *Rcm6* region. These genes were subjected to the sequence variation analysis using the genome data from five susceptible (E6203, Heinz 1706, Moneymaker, Ailsa Craig, and Black Cherry) and two resistant (Hawaii 7998 and Hawaii 7996) cultivars. Seventeen candidate R-genes harboring amino acid variations between the susceptible and resistant cultivars were identified and nine candidate genes contained putative functional mutations (Table 2). To validate the sequence variations identified using public genome data, coding sequences of nine candidate genes were manually sequenced from the five susceptible and two resistant cultivars using primer sets in Supplementary Table 1. The amino acid sequence alignment of the nine candidate genes is presented in Supplementary Figure S7.

**Table 2:** List of potential candidate genes for bacterial canker resistance underlying *Rcm6.*

Candidate gene	Position (bp)	Nucleotide change^a^		Amino acid change		Description
		Susceptible	Resistant	Susceptible	Resistant	
Solyc06g060680.2.1	38824367	TGGAGGTAAT	-	L	frameshift	Receptor-like kinase (RLK)
Solyc06g060690.2.1	38828575	C	T	P	S	Receptor-like kinase (RLK)
	38828983	T	A	S	T	
Solyc06g060700.1.1	38835047	TCTCCTCTGCTTG	G-	L	frameshift	Protein kinase
Solyc06g062440.3.1	39509899	A	G	S	G	Nucleotide-binding domain leucine-rich repeat (NLR)
Solyc06g062450.3.1	39521478	C	T	L	F	Receptor-like kinase (RLK)
Solyc06g063150.3.1	40008352	G	A	C	Y	Receptor-like kinase (RLK)
	40008545	A	G	T	A	
Solyc06g064680.1.1	40431793	G	A	V	I	Nucleotide-binding domain leucine-rich repeat (NLR)
	40431861	A	T	E	D	
	40433251	T	G	F	V	
	40433446	T	G	S	A	
Solyc06g064710.1.1	40448680	C	G	T	R	Nucleotide-binding domain leucine-rich repeat (NLR)
	40448908	T	A	F	Y	
	40448943	A	G	N	D	
Solyc06g064720.1.1	40452607	T	A	N	K	Nucleotide-binding domain leucine-rich repeat (NLR)
	40452614	C	G	Q	E	
	40452627	A	T	K	M	
	40452739	T	A	N	K	
	40452755	A	G	T	A	
	40452776	G	T	A	S	
	40452824	GAC	TAT	D	Y	
	40452828	A	G	D	G	
	40452876	A	G	K	R	
	40453238	A	G	K	E	
	40453500	C	T	A	V	
	40454342	G	T	D	Y	
	40454355	T	G	F	C	
Solyc06g064750.1.1	40466829	ATA	GTG	I	V	Nucleotide-binding domain leucine-rich repeat (NLR)
	40466835	AGCATCTTC	-	HLQ	-	
	40466892	G	A	E	K	
	40466902	G	A	G	E	
	40466919	A	G	I	V	
	40467213	TT	CA	L	Q	
Solyc06g064760.1.1	40480599	G	C	E	D	Nucleotide-binding domain leucine-rich repeat (NLR)
	40481125	C	G	H	D	
	40481243	T	A	L	Q	
	40481247	C	A	H	Q	
	40481302	A	G	K	E	
	40481380	A	T	I	L	
	40481395	C	G	H	D	
	40481406	T	A	D	E	
	40481473	G	C	D	H	
	40481480	TC	AG	L	Q	
	40481556	G	C	L	F	
	40481561	G	A	G	D	
	40481573	A	G	K	R	
	40481586	G	A	M	I	
	40481590	TA	GT	Y	V	
	40481593	GGA	TCC	G	S	
	40481596	A	G	R	G	
	40481598	-	AATGA	N	frameshift	
Solyc06g064790.1.1	40494375	G	C	L	F	Nucleotide-binding domain leucine-rich repeat (NLR)
	40495272	G	A	D	N	
Solyc06g065000.2.1	40652563	T	A	K	M	Nucleotide-binding domain leucine-rich repeat (NLR)
	40652692	T	C	E	G	
	40652563	T	A	K	M	
	40652692	T	C	E	G	
Solyc06g065120.1.1	40740430	-	T	*	I	Nucleotide-binding domain leucine-rich repeat (NLR)
Solyc06g065130.1.1	40742553	ATCCTCATCC	-	Y	*	Nucleotide-binding domain leucine-rich repeat (NLR)
Solyc06g065150.1.1	40746042	C	T	L	F	Receptor-like protein (RLP)
	40746148	A	G	K	R	
	40746508	A	G	N	S	
	40746513	-	A	Y	*	
Solyc06g065260.3.1	40819050	A	C	E	D	Receptor-like kinase (RLK)

a Nucleotide variation between susceptible (Heinz 1706, E6203, Moneymaker, Ailsa Craig, and Black Cherry) and resistant (Hawaii 7996 and Hawaii 7998) lines. Mutations within the putative functional domains are highlighted in gray. Hyphen indicates a deletion and asterisk indicates a stop codon.

The deletion of 10 nucleotides in Solyc06g060680.2.1 (RLK) resulted in the deletion of 146 amino acids containing putative protein kinase domain from the resistant cultivars. Eight candidate genes: Solyc06g060690.2.1 (RLK), Solyc06g062450.3.1 (RLK), Solyc06g063150.3.1 (RLK), Solyc06g064680.1.1 (NLR), Solyc06g064720.1.1 (NLR), Solyc06g064750.1.1 (NLR), Solyc06g064760.1.1 (NLR), and Solyc06g065150.1.1 (RLP) contained amino acid substitutions between susceptible and resistant cultivars within the putative functional domains. Solyc06g060690.2.1 (RLK) contained Ser132Thr and Pro237Ser in the protein kinase domain. Solyc06g062450.3.1 (RLK) and Solyc06g063150.3.1 (RLK) harbored Leu6Phe and Cys271Try in the transmembrane domain. Solyc06g064680.1.1 (NLR) harbored Val121Ile and Glu143Asp in the NB-ARC domain. Solyc06g064720.1.1 (NLR) contained Asn37Lys, Gln40Glu, Lys44Met, Asn81Lys, Thr87Ala, Ala94Ser, Asp110Tyr, Lys127Arg, and Lys248Glu in the NB-ARC domain. Solyc06g064750.1.1 (NLR) harbors Ile174Val, Glu195Lys, Gly198Glu, Ile204Val, and Leu302Gln in the NB-ARC domain. In addition, Solyc06g064750.1.1 harbored the deletion of His-Leu-Gln from the resistant cultivars within the NB-ARC domain. Solyc06g064760.1.1 (NLR) contained His181Asp, Leu220Gln, His221Gln, Lys240Glu, Ile266Leu, His271Asp, Asp274Glu, Asp297His, and Leu299Gln in the NB-ARC domain. Solyc06g065150.1.1 (RLP) contained Leu32Phe in the LRR domain (Table 2; Supplementary Figure S7).

The expression level of nine candidate genes harboring putative functional mutations was analyszed upon the *Cm* infection at 0, 2, and 4 dpi in leaf tissues of “E6203” and “Hawaii 7998” by qRT-PCR. The expression of Solyc06g060680.2.1, Solyc06g060690.2.1, and Solyc06g064750.1.1 was downregulated while the expression of Solyc06g064720.1.1, Solyc06g064760.1.1, and Solyc06g065150.1.1 was significantly upregulated after the *Cm* infection in “E6203.” The expression of Solyc06g063150.3.1 and Solyc06g064680.1.1 was not significantly changed upon the infection in both lines. The expression of Solyc06g062450.3.1 was downregulated while the expression of other candidate genes was not altered after the *Cm* infection in “Hawaii 7998” (Supplementary Figure S8).

## Discussion

Tomato bacterial canker is one of the devastating diseases causing substantial economic loss (Chang et al., 1992; Poysa, 1993). Breeding of a resistant cultivar is the most efficient and eco-friendly method to control tomato bacterial canker (Stüwe and von Tiedemann, 2013; Sen et al., 2015). The QTL analysis was conducted in a few resistant accessions of wild species for bacterial canker resistance. In *S. arcanum* “LA2157,” several QTLs were identified using backcross, F_2_, and RIL populations (Sandbrink et al., 1995; van Heusden et al., 1999; Sen, 2014). Although the same resistant parent was used, overlapping QTL was not identified in these studies. Such disparity might be due to differences in the susceptible parent, type of mapping population, environmental condition, inoculation methods, pathogen isolate, and threshold values for QTL detection (van Heusden et al., 1999). The QTLs in chromosomes 2 and 5, which showed an additive effect, were reported in *S. habrochaites* “LA407” (Kabelka et al., 2002; Coaker and Francis, 2004). Some studies showed that bacterial canker resistance is controlled by a single dominant gene while most of the other studies reported polygenic resistance as previously reviewed (Wang et al., 2018). A single recessive resistance gene was proposed in *S. arcanum* “LA2157” (Sen, 2014).

Genetic analysis of bacterial canker resistance was majorly focused on wild species (Sandbrink et al., 1995; van Heusden et al., 1999; Kabelka et al., 2002; Coaker and Francis, 2004; Sen, 2014) and was not conducted in *S. lycopersicum*, although the resistance was found in *S. lycopersicum* “Hawaii 7998,” “IRAT L-3,” “Okitsu Sozai I-20,” and “Bulgaria 12” (Steekelenburg, 1985; Gardner et al., 1990; Poysa, 1993). The genetic basis of the resistance in cultivated tomatoes remains uncovered. The genetic analysis of resistance in “Hawaii 7998” will minimize a linkage drag, cross-incompatibility and hybrid sterility, which are major drawbacks using wild species as a resistance source (Dempewolf et al., 2017).

In tomato, QTL-seq was successfully conducted to identify genomic regions associated with gray leaf spot (Yang et al., 2017), heat tolerance (Wen et al., 2019), fruit weight and locule number (Illa-Berenguer et al., 2015), and early flowering (Ruangrak et al., 2018). In this study, the QTL-seq was used to identify genomic regions for bacterial canker resistance in “Hawaii 7998.” The susceptible and resistant F_2_ bulks from two independent experiments were used for QTL-seq analysis to identify a significant QTL. Furthermore, to increase the efficiency and accuracy of mapping, resistant and susceptible parent genome assemblies were used as a reference for mapping the reads of the F_2_ bulk sequences (Luo et al., 2019). The *Rcm6* was commonly identified in experiments I and II and located between 37.24 (Rcm6-1) and 41.15 (Rcm6-12) Mb on chromosome 6 (Figures 2A,B). One major QTL linked with the *Rcm6* was identified in *S. arcanum* “LA2157” using a RIL population (Sen, 2014). The *Rcm6* was dissected with 12 InDel markers in 47 cultivars (Table 1). Two *Rcm6* regions (Rcm6-2–Rcm6-4 and Rcm6-8–Rcm6-9) showed the association to the resistance in the tomato cultivars (Table 1). The F_2_ and F_3_ progenies harboring “Hawaii 7998” alleles to Rcm6-9 showed increased resistance to bacterial canker compared to those harboring “E6203” alleles (Figures 3B,C). The potential of Rcm6-9 for marker-assisted selection was validated in diverse tomato cultivars, F_2_ population, and F_3_ progenies, and could efficiently develop elite cultivars with enhanced resistance. Taken together, these results indicate that *Rcm6* is a resistance locus of bacterial canker in *S. lycopersicum*. The marker-assisted breeding for tomato disease resistance is well-established (Lee et al., 2015), and Rcm6-9 will be exploited since a resistant cultivar against the bacterial canker is not commercially available yet.

Candidate genes for *Rcm6* were mined based on the previously identified R-genes (Kourelis and van der Hoorn, 2018; Zhang et al., 2019; Deng et al., 2020). The RLK/RLPs (extracellular) and NLRs (intracellular) receptors are well-known R-genes against diverse pathogen effectors (Kourelis and van der Hoorn, 2018). Nine candidate genes encoding RLK/RLPs or NLRs in the *Rcm6* were identified to harbor putative functional mutations. The RLK/RLPs and NLRs act as receptors that recognize pathogen effectors directly or indirectly and initiate resistance response (Tang et al., 2017; Deng et al., 2020).

Receptor-like kinase genes conferring resistance to diverse plant pathogens have been identified in different plant species as previously reveiwed (Yang et al., 2012; Liang and Zhou, 2018). RLK candidate genes Solyc06g060680.2.1 and Solyc06g060690.2.1 harbored 146 amino acid deletion and two amino acid substitutions in the protein kinase domain, respectively. RLK candidate genes Solyc06g062450.3.1 and Solyc06g063150.3.1 contain single amino acid substitutions in the transmembrane domain. Single amino acid susbstitution in the transmembrane domain of *PYRICULARIA ORYZAE RESISTANCE D 2* (*Pi-d2*; RLK) resulted in resistance to rice blast caused by the fungal pathogen *Magnaporthe grisea* (Li et al., 2015). The NB-ARC domain of NLR proteins is proposed as a molecular switch which regulates defense response (Takken et al., 2006; van Ooijen et al., 2007). Amino acid substitutions in the NB-ARC domain of NLR proteins resulted in a loss or gain of function phenotype in many R-proteins (DeYoung and Innes, 2006). NLR candidate genes, Solyc06g064680.1.1, Solyc06g064720.1.1, Solyc06g064750.1.1, and Solyc06g064760.1.1, harbor amino acid substitutions in the NB-ARC domain (Table 2; Supplementary Figure S7). Amino acid substitutions in the NB-ARC domain of tomato *I-2* resulted in impaired ATP hydrolysis and autoactivation of defense response. In addition, the protein displayed an increased affinity to ADP, which might be due to conformational change (Tameling et al., 2006). It is also shown that tomato *I-2* and *Mi-1* are able to bind ATP and exert ATPase activity, which are thought to be the general features of NLR encoding proteins. The *I-2* mutant harboring amino acid substitution in the NB-ARC domain showed reduced ATP binding and hydrolysis (Tameling et al., 2002). *MELOIDOGYYNE INCOGNITA-1.2* (NLR) harboring amino acid substations in the NB-ARC domain confer resistance to tomato root-knot nematode (van Ooijen et al., 2008). *DOMINANT SUPRESSOR OF CAMTA3 NUMBER 1* (NLR) harboring single amino acid substitution in the NB-ARC domain confer resistance against Verticillium wilt in cotton (Li et al., 2019). Single amino acid substitution in the NB-ARC domain of tomato NRC1 (NB-LRR Required for Hypersensitive Response-Associated Cell Death-1) was associated with induction of the elicitor-independent hypersensitive response in *Nicotiana tabacum* (Sueldo et al., 2015). Single amino acid substitution in the NB-ARC domain of *Os11g0646300* (NLR) led to the disruption of immunity against bacterial blight in rice (Tang et al., 2019). Solyc06g065150.1.1 (RLP) harbored a single amino acid substitution in the LRR domain. Single amino acid substitution in the LRR domain of *SPOTTED LEAF 36* (RLK) in rice resulted in resistance to bacterial blight (Rao et al., 2021). The mutations in the candidate genes may cause conformational change of the respective proteins, thereby affecting downstream signaling pathways in response to *Cm* infection. Taken together, these mutations in the candidate genes may control the bacterial canker resistance.

*Cm* infection caused differential gene expression including defense and stress responsive genes in tomato (Balaji et al., 2008; Lara-Ávila et al., 2012; Hwang et al., 2020; Basim et al., 2021). Expression profiles of nine candidate genes harboring putative functional mutations were investigated by qRT-PCR to better understand if their expression is associated to *Cm* resistance. The expression of Solyc06g060680.2.1 (RLK), Solyc06g060690.2.1 (RLK), Solyc06g064720.1.1 (NLR), Solyc06g064750.1.1 (NLR), Solyc06g064760.1.1 (NLR), and Solyc06g065150.1.1 (RLP) was significantly altered in “E6203” while their expression was not changed in ‘Hawaii 7998’ after the *Cm* infection. The expression of Solyc06g060680.2.1 (RLK), Solyc06g060690.2.1 (RLK), and Solyc06g064750.1.1 (NLR) was downregulated in “E6203” after *Cm* infection, and these genes may be involved in positive regulation of basal defense responses. The expression of Solyc06g064720.1.1 (NLR), and Solyc06g064760.1.1 (NLR), and Solyc06g065150.1.1 (RLP) was significantly upregulated after *Cm* infection in “E6203,” hence these genes may be involved in defense response independent of effector-triggered immunity. Solyc06g062450.3.1 (RLK) was significantly downregulated in “Hawaii 7998” after *Cm* infection indicating that this gene might be involved in negative regulation of *Cm* resistance. Many RLK/RLP are known to be involved in negative regulation of plant innate immunity (Yang et al., 2012). The *SPOTTED LEAF 36* (RLK) negatively regulates resistance to bacterial blight of rice by downregulating the expression of defense related genes (Rao et al., 2021). Hence, it is plausible that Solyc06g062450.3.1 (RLK) can negatively regulate downstream defense response to the *Cm* infection. Further functional analysis of the candidate genes will be required to define the R-gene and the underlying mechanism regulating the *Cm* resistance in tomato.

## Data Availability Statement

The sequencing raw datasets generated in this study have been deposited in the NCBI Sequence Read Archieve (https://www.ncbi.nlm.nih.gov/bioproject/) under BioProject accession number PRJNA704807.

## Author Contributions

JML conceived and designed the experiments. AMA, HTK, GC, and ES performed the experiments. CSO and IY provided experimental materials. AMA and JML wrote the manuscript. All authors contributed to the article and approved the submitted version.

## Funding

This work was supported by the Golden Seed Project (#213007-05-3-CGF00, Center for Horticultural Seed Development) funded by the Ministry of Agriculture, Food and Rural Affairs of Korea.

## Conflict of Interest

The authors declare that the research was conducted in the absence of any commercial or financial relationships that could be construed as a potential conflict of interest.

## Publisher’s Note

All claims expressed in this article are solely those of the authors and do not necessarily represent those of their affiliated organizations, or those of the publisher, the editors and the reviewers. Any product that may be evaluated in this article, or claim that may be made by its manufacturer, is not guaranteed or endorsed by the publisher.

## Footnotes

^1^ http://pfam.janelia.org/

^2^ http://smart.embl-heidelberg.de/
