# Supplementary material for: QTL-Seq Analysis for Identification of Resistance Loci to Bacterial Canker in Tomato
Source: Front Plant Sci. 2022 Jan 26;12:809959. doi: 10.3389/fpls.2021.809959 (PMC8826648; doi:10.3389/fpls.2021.809959)
Supplement: Supplementary file 1 [file Data_Sheet_1.docx]

**Supplementary Table 1** Primers used for candidate gene sequencing and qRT-PCR analysis

| Candidate gene | Primer sequence (5' - 3') | |
| --- | --- | --- |
|  | Forward | Reverse |
| **For candidate gene sequencing** | | |
| Solyc06g060680.2.1 | TCATTTTGAG CAAGAGCGAA | ATGGAGCTCCGAGGTGTGGTTTT |
| Solyc06g060690.2.1 | TTTCCACTGATGCTACCTAC | ACCCGAGTCTTCTCCAAAAT |
|  | ATGTCTACAGCTTTGGGATT | GAATGTGCTGCTTCA ATC AA |
| Solyc06g062450.3.1 | ATGTTGAAAGGCTTCCTCTT | TTCTTAAGATCGATCTGCCT |
|  | TTCCTTAACCAATCAGTACG | GCTTTCATCCACATAGATGA |
| Solyc06g063150.3.1 | TCAGTCCTGATCATTATTGTG | TGTGTGTGTTTTCTCTTCTC |
|  | TGAGTGGAGAAGAGAAAACA | ACCTCTCCAATTCTTATGAG |
|  | CCGGTGTTCTACA ACTTTAA | CATTTGCCCTTTTCTAGTCA |
|  | GACCCAAAAAAGAAGTCACA | ATGCAGTGAGTGTTTCAGTT |
|  | AACTGAAACA CTCACTGCAT | CCTTGTTGCTTACGTTTGAT |
|  | AACTGAAACACTCACTGCAT | CCTTGTTGCTTACGTTTGAT |
|  | ACGGGACAAATTGAAACAAG | GCTGATTTTCACTTTTCGTT |
|  | AATATTG GGTTCAGTTGAAC | AGGAAAATGAAACGAAAAGTG |
| Solyc06g064680.1.1 | ATGGATGTTGTTGAGACTTG | AAATCACTCAAGCAAACTCG |
|  | GCTTAACAGAAGCCTAATAA | TTACTTCTGTATAACAACTTGG |
| Solyc06g064720.1.1 | ATGGAAGTTG TTGAGACTTG | GCTTTTGTTAAGCAAATCAC |
|  | AAGCATATCGAGTTTGCTTG | ACTTGTTTCAAAACCTCCTTC |
|  | AATTCAATCACTAACTGAGTTGAGG | TTATTTTGGTTTTACAACTACAACATGG |
| Solyc06g064750.1.1 | ATGGAGGTTGTTGGAACTTGT | AAGACAACCGTAAGAGGCAAT |
|  | ATCTAGTGGAAGCAGGGTTA | TGAGCAATGAACTCCAAAGA |
|  | TCCAAAACTCAATGTGTGGA | AAACATTTGGCAAGGTCCAA |
|  | CCCTTCAAATCTTAGGCATT | TTACTTCTGTATAACAACTTGG |
| Solyc06g064760.1.1 | ATGGAAGTTGTTGAGACTTG | CATGTTCTCTGTTTCAGAAT |
|  | TTTACATGGCATTGTTTCCA | TCATGTTCTCTAGCATACTT |
|  | AAATGAAGAAGCTAAGGCAT | TTATTTATGTTTTTCCATTAGAACACG |
| Solyc06g065150.1.1 | ATGTTACCCTCTTCCTGGGA | TCAAGACTTCCGTATTTTTTTTG |
| **For qRT-PCR analysis** | | |
| Solyc06g060680.2.1 | GCTCCAGAATATGAGCAGCAG | CATAACGATCCACGTTGGTTGC |
| Solyc06g060690.2.1 | CGCGTTTCCACTGATGCTAC | GGCACATCGCTACTCACCAG |
| Solyc06g062450.3.1 | ACGGCGTTGATACCTGATCC | AGGCAGGAGACAGAGGTCCA |
| Solyc06g063150.3.1 | CCTCGCCAATCTTCCTCTTG | AGGCTCTGAACTCCCTGTG |
| Solyc06g064680.1.1 | GCCTAATAATAATGGTCTCCGG | TGCATAACCGTGACTCTGTG |
| Solyc06g064720.1.1 | CTGAGGTTCTCAGTAATGGAAG | CCAATAATGGTACTCTGATCTCC |
| Solyc06g064750.1.1 | GAAGCCTAATAATGGTCTCCGG | CAACCGTGATATCGTGGAATGC |
| Solyc06g064760.1.1 | TCTTAGATTCAGTTGGATGGG | TTGTCACATCCCAGCATAAGC |
| Solyc06g065150.1.1 | GTGCAATTCCCACCTGGTTT | CAACACACCTGCTCCCATGT |
| *GAPDH* | CTGGTGCTGACTTCGTTGTTG | GCTCTGGCTTGTATTCATTCTCG |

**Supplementary Table 2** Summary of whole-genome resequencing for the parental lines and F_2_ bulks

| Sample ID | Before trimming | | After trimming | | Genome Coverage (X) | GC (%) | Q20 (%) | Q30 (%) |
| --- | --- | --- | --- | --- | --- | --- | --- | --- |
|  | Total read  bases (bp) | Total reads | Total read  bases (bp) | Total reads |  |  |  |  |
| Hawaii 7998 | 32,212,398,744 | 213,327,144 | 26,477,998,840 | 186,352,986 | 35.79 | 35.81 | 94.82 | 90.01 |
| E6203 | 10,578,365,098 | 70,055,398 | 9,179,782,168 | 63,832,968 | 11.75 | 35.1 | 96.04 | 91.21 |
| S-bulk1 | 10,662,082,518 | 70,609,818 | 9,197,459,757 | 64,045,990 | 11.84 | 35.76 | 95.88 | 90.92 |
| R-bulk1 | 10,396,110,212 | 68,848,412 | 8,977,452,657 | 62,533,046 | 11.55 | 35.88 | 95.88 | 90.96 |
| S-bulk2 | 38,020,737,564 | 251,792,964 | 34,402,069,625 | 235,233,872 | 42.24 | 35.87 | 97.31 | 93.94 |
| R-bulk2 | 35,613,605,794 | 235,851,694 | 32,249,143,490 | 220,468,580 | 39.57 | 35.83 | 97.33 | 94.00 |

**Supplementary Table 3** Primer information of InDel markers for dissecting the *Rcm6*

| Marker Name | Physical position (bp) | InDel size (bp) | Primer sequence (5’-3’) | | Tm (**°**C) | Product size (bp) | |
| --- | --- | --- | --- | --- | --- | --- | --- |
|  |  |  | Forward | Reverse |  | E6203 | Hawaii 7998 |
| Rcm6-1 | 37244888 | 50 | AATATTTATGATGTCCACGCAGAC | GCTCCCAATTTTAGCATCATTTAC | 57.5 | 225 | 175 |
| Rcm6-2 | 37250054 | 44 | TCTATTGAATGATCAGAACTTGGG | GACCTCGGAATCACTATTATTTAC | 57.5 | 186 | 142 |
| Rcm6-3 | 37698049 | 50 | TTCGTTTTTATTCAGTTCTCTGCC | AAAGGAAATTGACGGCAGTAAAAG | 57.5 | 232 | 186 |
| Rcm6-4 | 38147477 | 48 | TTGTGATAAAGGAATCGTATGGTC | GTTGATACAAGAAACCATCACTAC | 57.5 | 130 | 178 |
| Rcm6-5 | 38738773 | 71 | ATTCATATGCTCACCCATATACTC | GTACGATTGTTTCTCTACCTATTG | 57.5 | 287 | 358 |
| Rcm6-6 | 39133340 | 54 | CATTCTCAAATATTGGACAAGGAC | AAACTTAAGAACTCTGGACCAAAC | 57.5 | 173 | 227 |
| Rcm6-7 | 39450945 | 44 | GTTCAAAAACCCCCGAATAATATC | TATCATACTTGTGTATTTTGCGGC | 57.5 | 199 | 156 |
| Rcm6-8 | 39786266 | 30 | AATGATCAGAGCATATATGCAAGG | TGCATATGTCTATCATGCAACAAG | 57.5 | 192 | 222 |
| Rcm6-9 | 40054464 | 48 | CCTTCAATACTTGATTGAC | TTATCCCTAGATTGTAGTG | 50.1 | 242 | 194 |
| Rcm6-10 | 40607278 | 79 | CTTACACACTCGAATTTCACAATG | GTGAAGCTTAACGTTAGTTAATCC | 57.5 | 348 | 269 |
| Rcm6-11 | 41071431 | 59 | CATCAGAACAATGACTTAATACAC | GAGATAAACATATCTTAGAGTTTCAG | 57.5 | 317 | 258 |
| Rcm6-12 | 41155692 | 67 | TGTAGTCAAATTGAAATTGGCTGC | TTATCCCCCTAACACTAGAATAAC | 57.5 | 175 | 242 |

**Supplementary Table 4** Summary of diagnostic accuracy of InDel markers for bacterial canker resistance in 11 resistant and 36 susceptible cultivars (listed in Table 1)

| Marker Name | Diagnostic test result^a^ | | | | | | | |
| --- | --- | --- | --- | --- | --- | --- | --- | --- |
|  | Number of true positives | Number of false negative | Number of true negative | Number of False positive | True positive rate (%) | False negative rate (%) | True negative rate (%) | False positive rate (%) |
| Rcm6-1 | 10 | 1 | 33 | 3 | 90.9 | 9.1 | 91.7 | 8.3 |
| Rcm6-2 | 10 | 1 | 36 | 0 | 90.9 | 9.1 | 100.0 | 0.0 |
| Rcm6-3 | 10 | 1 | 36 | 0 | 90.9 | 9.1 | 100.0 | 0.0 |
| Rcm6-4 | 10 | 1 | 36 | 0 | 90.9 | 9.1 | 100.0 | 0.0 |
| Rcm6-5 | 10 | 1 | 33 | 3 | 90.9 | 9.1 | 91.7 | 8.3 |
| Rcm6-6 | 10 | 1 | 34 | 2 | 90.9 | 9.1 | 94.4 | 5.6 |
| Rcm6-7 | 11 | 0 | 31 | 5 | 100.0 | 0.0 | 86.1 | 13.9 |
| Rcm6-8 | 10 | 1 | 36 | 0 | 90.9 | 9.1 | 100.0 | 0.0 |
| Rcm6-9 | 11 | 0 | 36 | 0 | 100.0 | 0.0 | 100.0 | 0.0 |
| Rcm6-10 | 10 | 1 | 34 | 2 | 90.9 | 9.1 | 94.4 | 5.6 |
| Rcm6-11 | 9 | 2 | 30 | 6 | 81.8 | 18.2 | 83.3 | 16.7 |
| Rcm6-12 | 10 | 1 | 30 | 6 | 90.9 | 9.1 | 83.3 | 16.7 |

^a^ True positive, Resistant cultivars with resistant genotype; False negative, Resistant cultivars with susceptible genotype; True negative, susceptible cultivars with susceptible genotype; False positive, susceptible cultivars with resistant genotype.

**
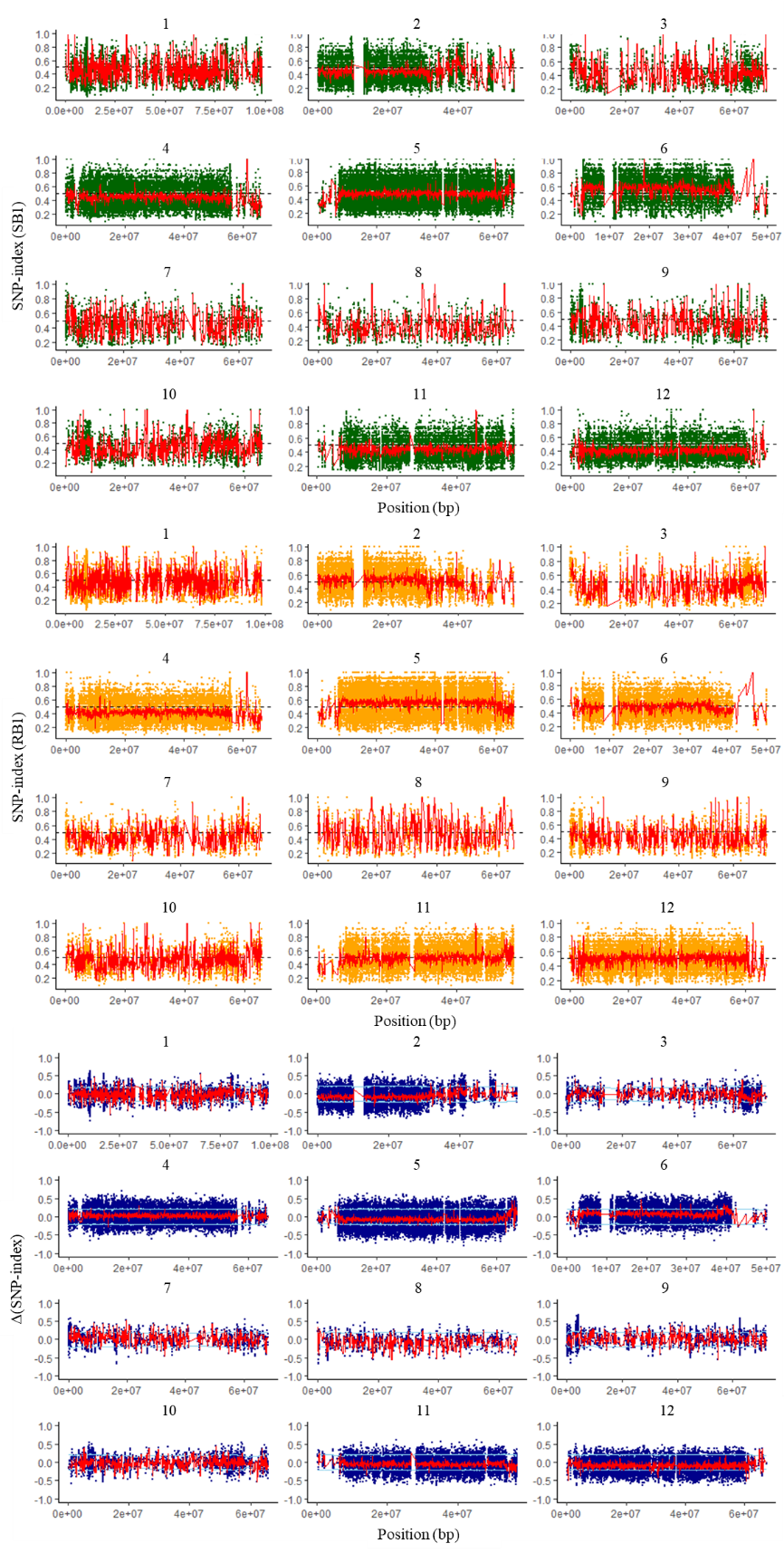
**

**Supplementary Figure 1** SNP-index plots for 12 chromosomes of resistant (orange), susceptible (dark green) bulks, and Δ(SNP-index) (dark blue) with ‘E6203’ as reference in experiment-I. Red lines indicate the sliding window average of 100 kb interval with 10 kb increment for SNP-index. Δ(SNP-index) plot was obtained by subtracting the susceptible bulk SNP-index from the resistant bulk SNP-index. Statistical confidence interval under the null hypothesis of no QTLs (*p* < 0.05) is indicated by light blue line.

**
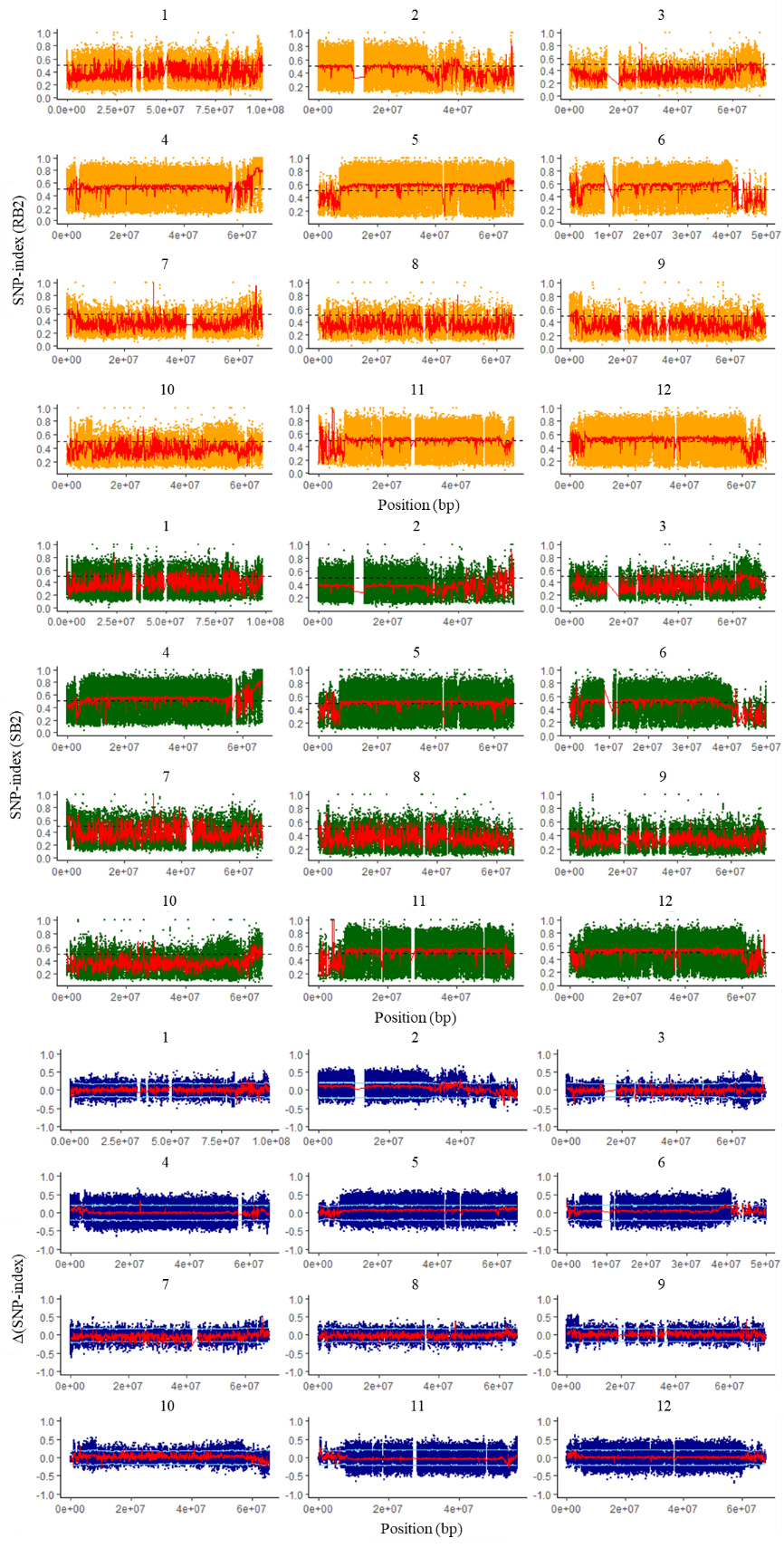
**

**Supplementary Figure 2** SNP-index plots for 12 chromosomes of resistant (orange), susceptible (dark green) bulks, and Δ(SNP-index) (dark blue) with ‘E6203’ as reference in experiment-II. Red lines indicate the sliding window average of 100 kb interval with 10 kb increment for SNP-index. Δ(SNP-index) plot was obtained by subtracting the susceptible bulk SNP-index from the resistant bulk SNP-index. Statistical confidence interval under the null hypothesis of no QTLs (*p* < 0.05) is indicated by light blue line.

**
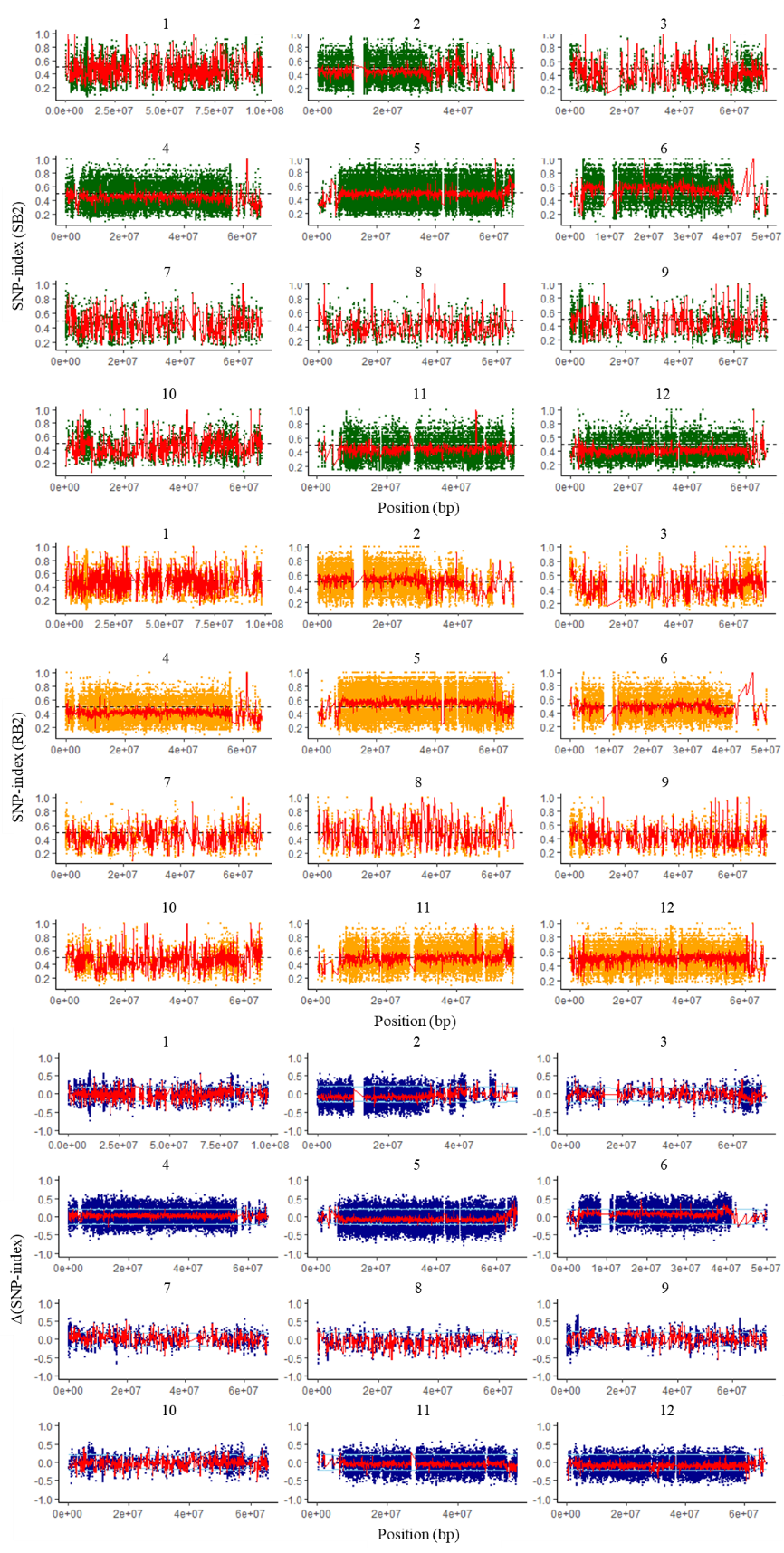
**

**Supplementary Figure 3** SNP-index plots for 12 chromosomes of the susceptible (dark green), resistant (orange) bulks, and Δ(SNP- index) (dark blue) with ‘Hawaii 7998’ as reference in experiment-I. Red lines indicate the sliding window average of 100 kb interval with 10 kb increment for SNP-index. Δ(SNP-index) plot was obtained by subtracting the resistant bulk SNP-index from the susceptible bulk SNP-index. Statistical confidence interval under the null hypothesis of no QTLs (*p* < 0.05) is indicated by light blue line.

**
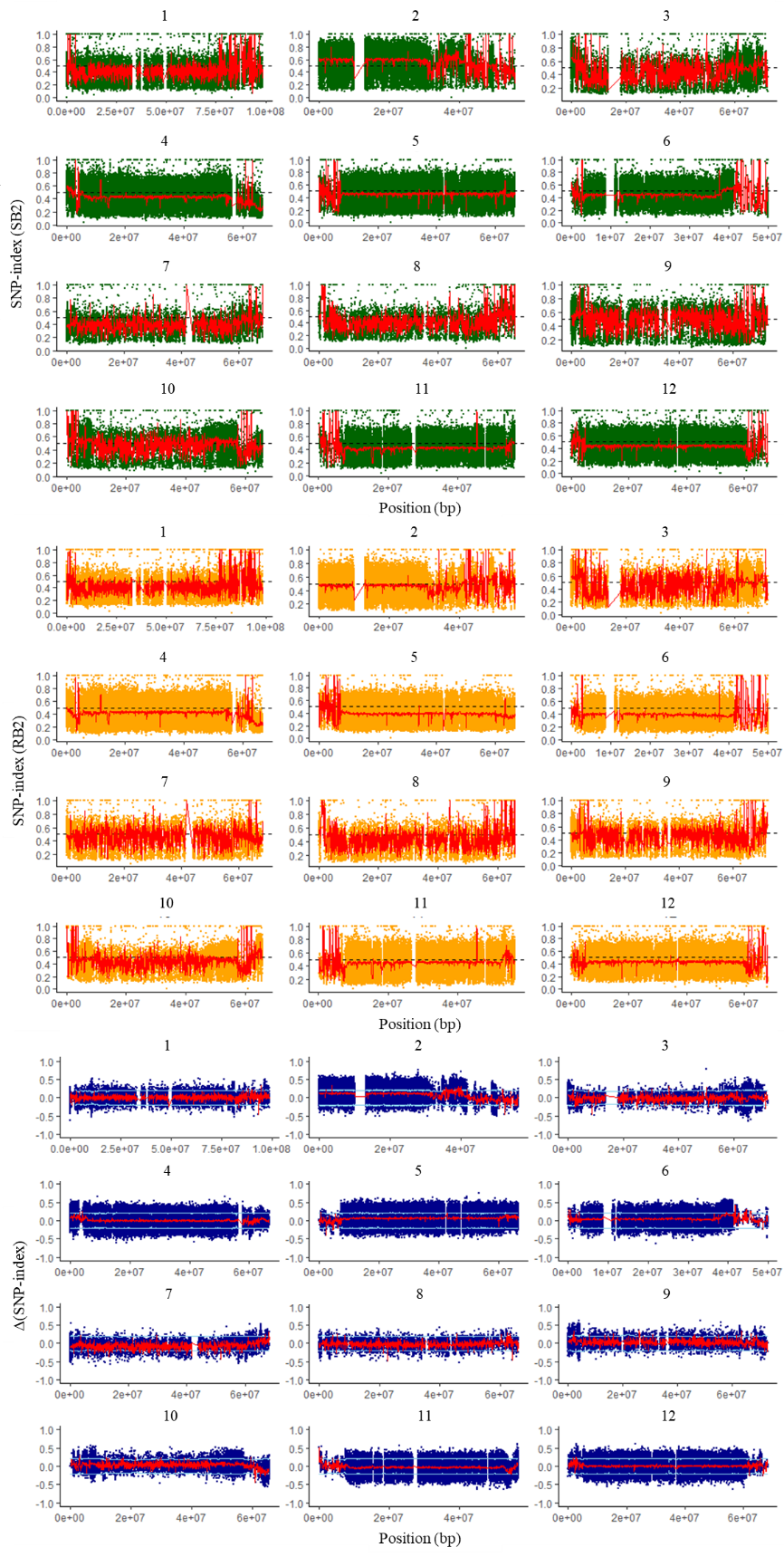
**

**Supplementary Figure 4** SNP-index plots for 12 chromosomes of the susceptible (dark green), resistant (orange) bulks, and Δ(SNP-index) (dark blue) with ‘Hawaii 7998’ as reference in experiment-I. Red lines indicate the sliding window average of 100 kb interval with 10 kb increment for SNP-index. Δ(SNP-index) plot was obtained by subtracting the resistant bulk SNP-index from the susceptible bulk SNP-index. Statistical confidence interval under the null hypothesis of no QTLs (*p* < 0.05) is indicated by light blue line.


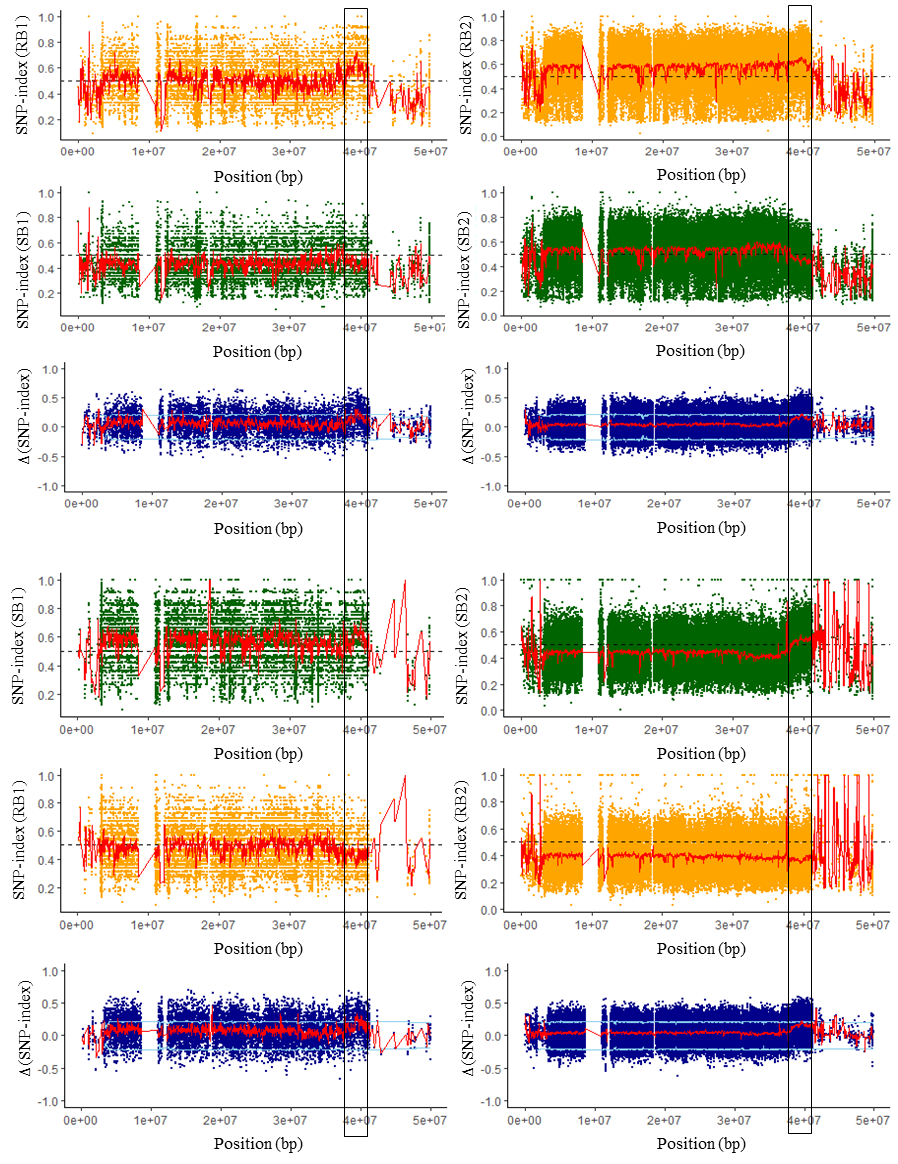


**Supplementary Figure 5** SNP-index plots for chromosome 6 of resistant (orange), susceptible (dark green) bulks, and Δ(SNP-index) (dark blue) with ‘E6203’ as reference (top) and ‘Hawaii 7998’ as reference (bottom) in experiment-I (left) and II (right). Red lines indicate the sliding window average of 100 kb interval with 10 kb increment for SNP-index. Black boxes indicate candidate genomic region for bacterial canker resistance. Light blue line indicates statistical confidence interval under the null hypothesis of no QTLs (*p* < 0.05).


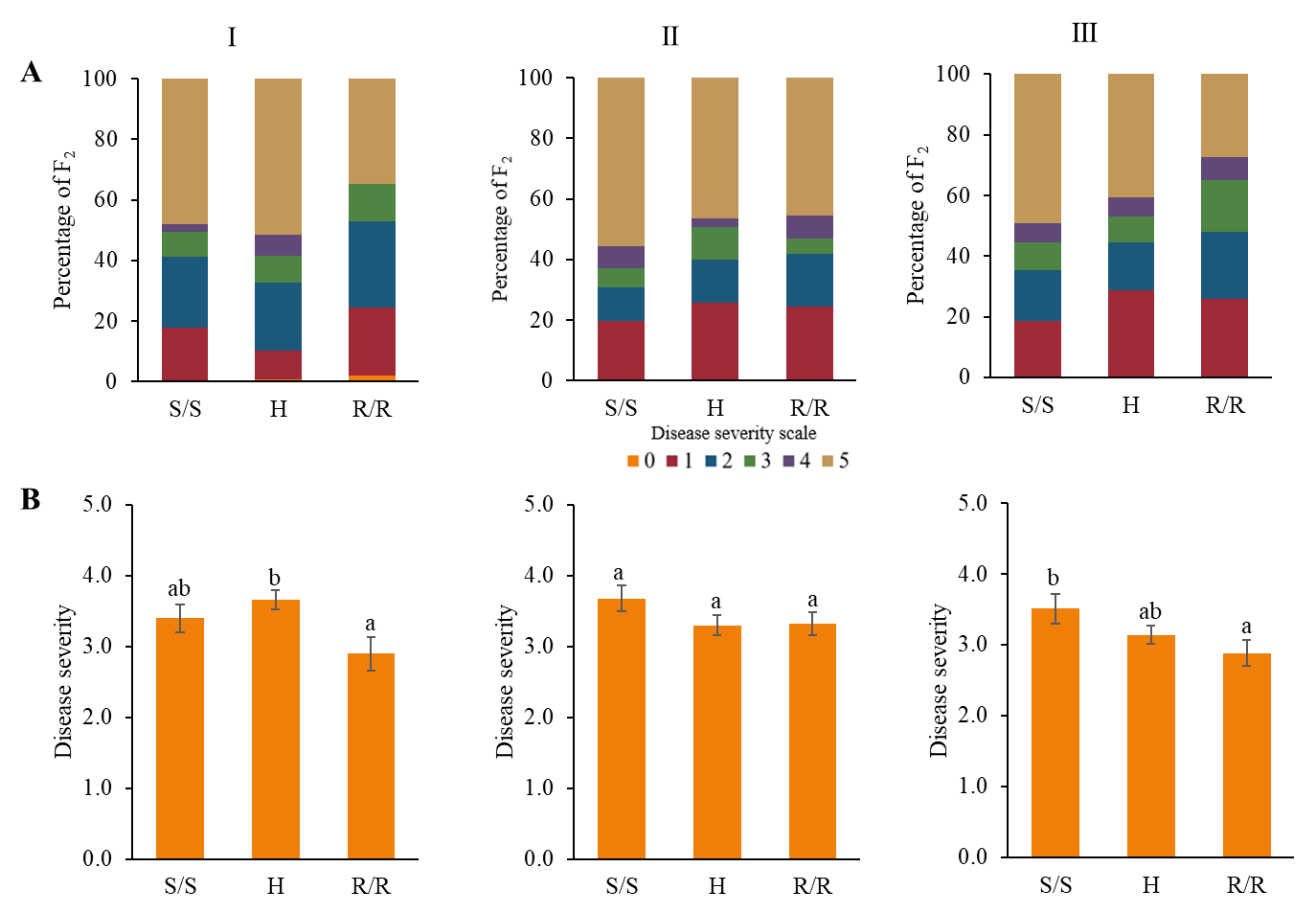


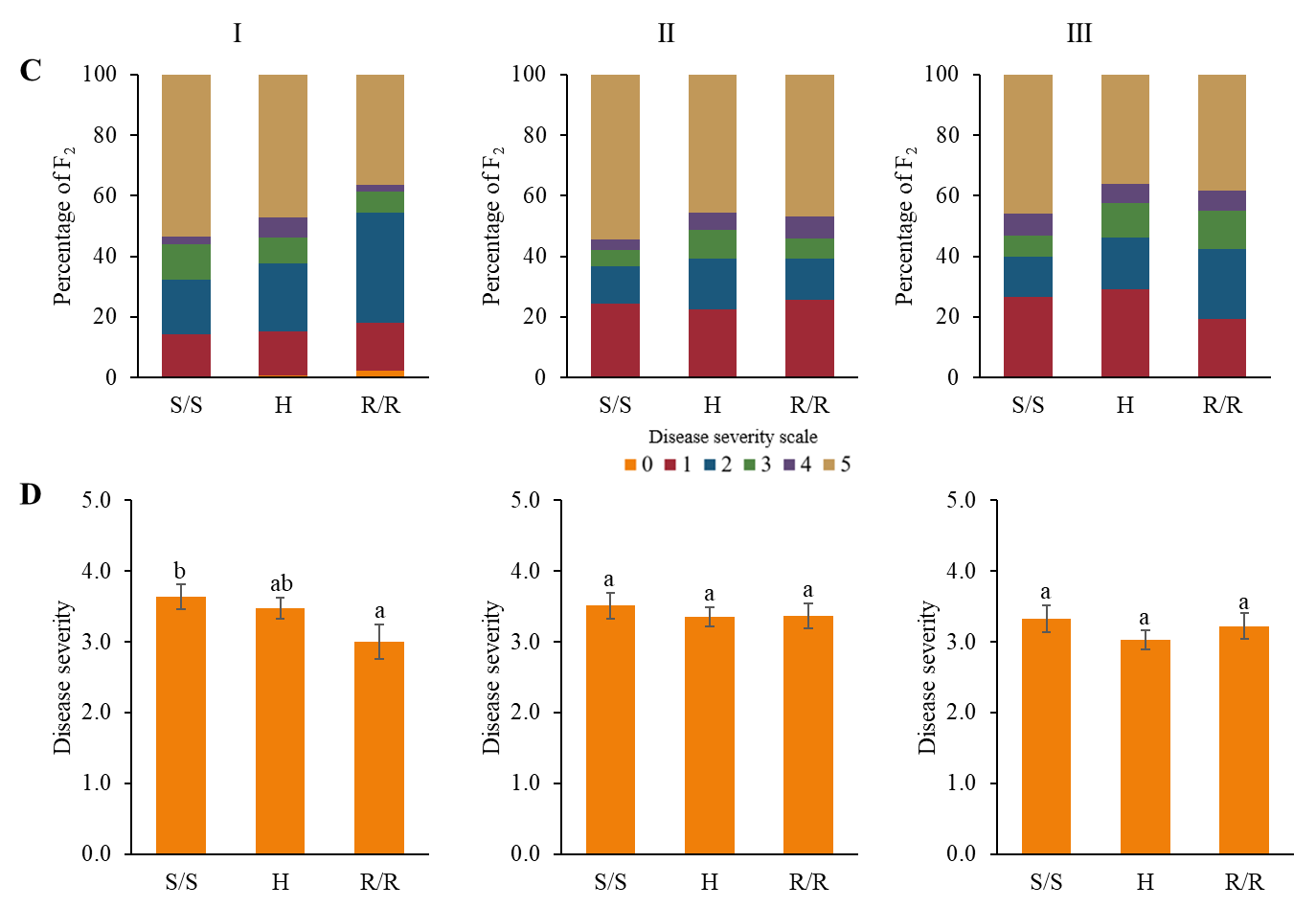


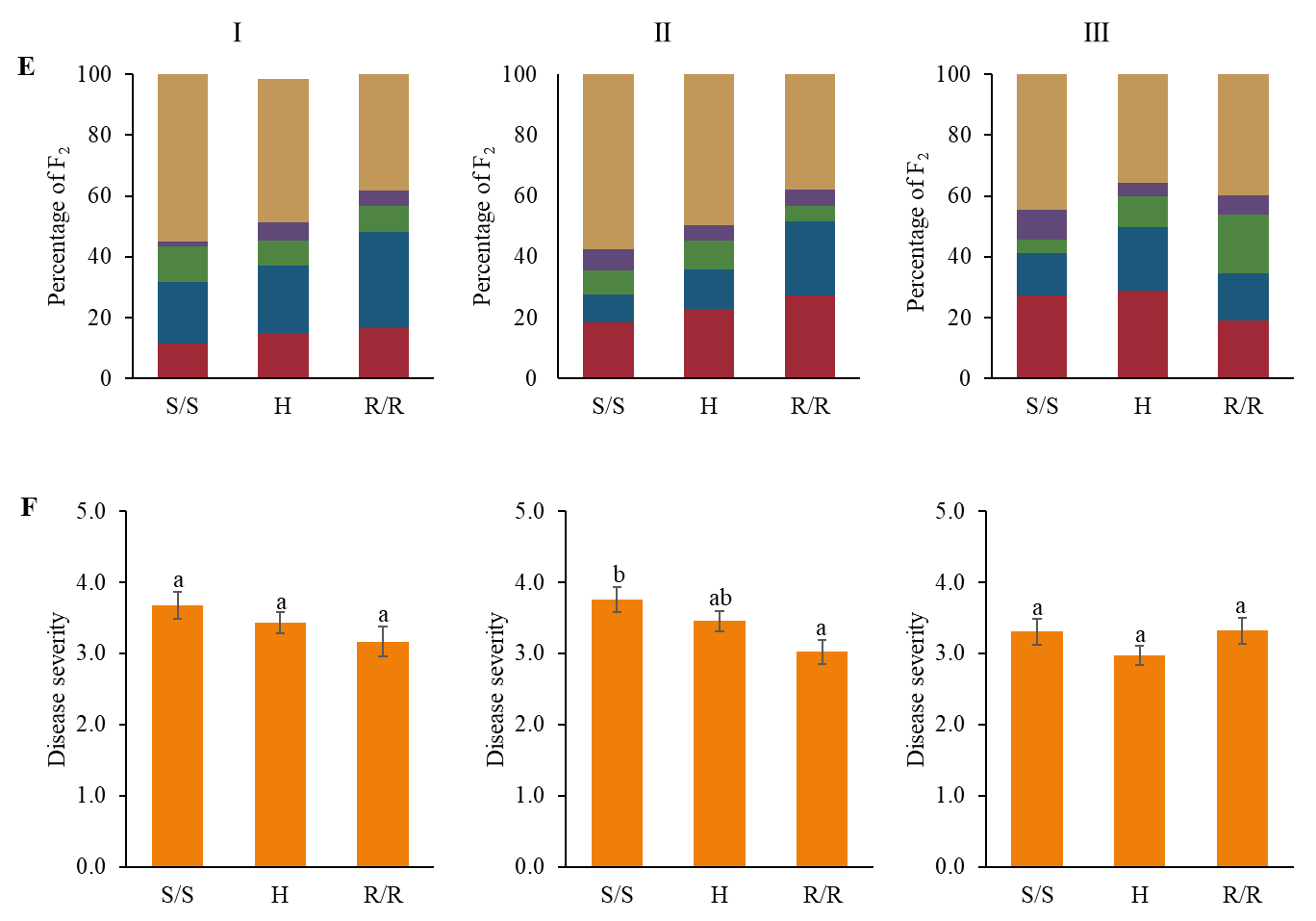


**Supplementary Figure 6** Performance of Rcm6-1 (**A** and **B**), Rcm6-5 (**C** and **D**), and Rcm6-12 (**E** and **F**) in the F_2_ population derived from ‘E6203’ and ‘Hawaii 7998’ in three experiments. **A, C,** and **E** percentage of F_2_ with each disease scale within the respective genotypes. **B, D,** and **F** mean values with different letters on the bars are significantly different (*p* < 0.05) according to Duncan’s multiple range test. S represents the susceptible ‘E6203’ allele, H represents the heterozygote, and R represents the resistant ‘Hawaii 7998’ allele.

Solyc06g060680.2.1 - Receptor-like kinase (RLK)


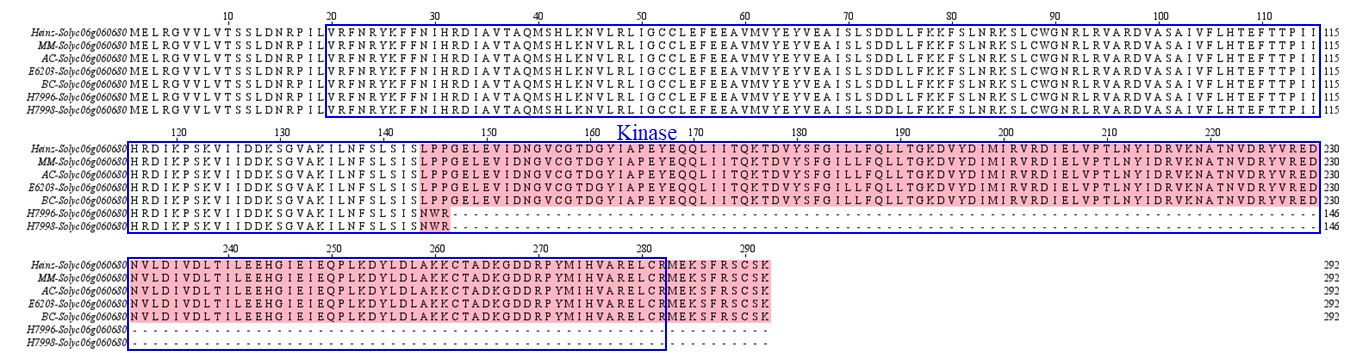


Solyc06g060690.2.1- Receptor-like kinase (RLK)


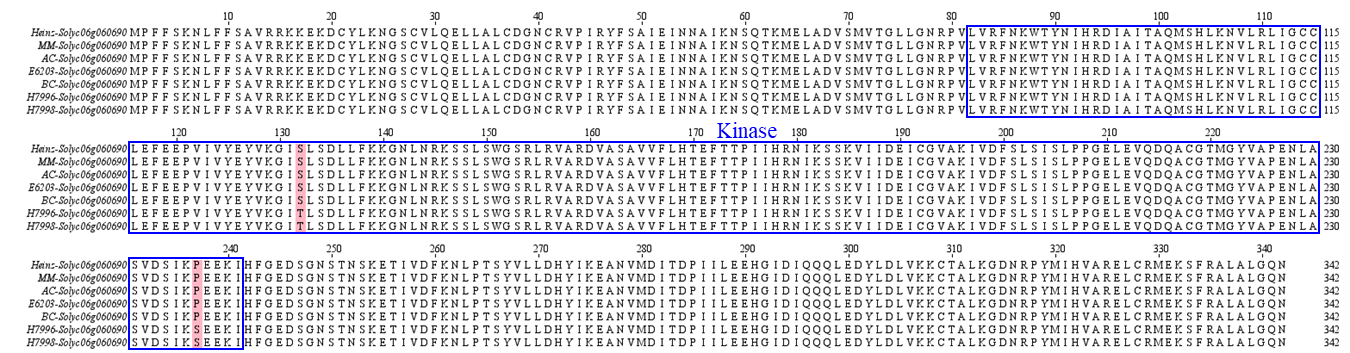


Solyc06g062450.3.1 – Receptor-like kinase (RLK)


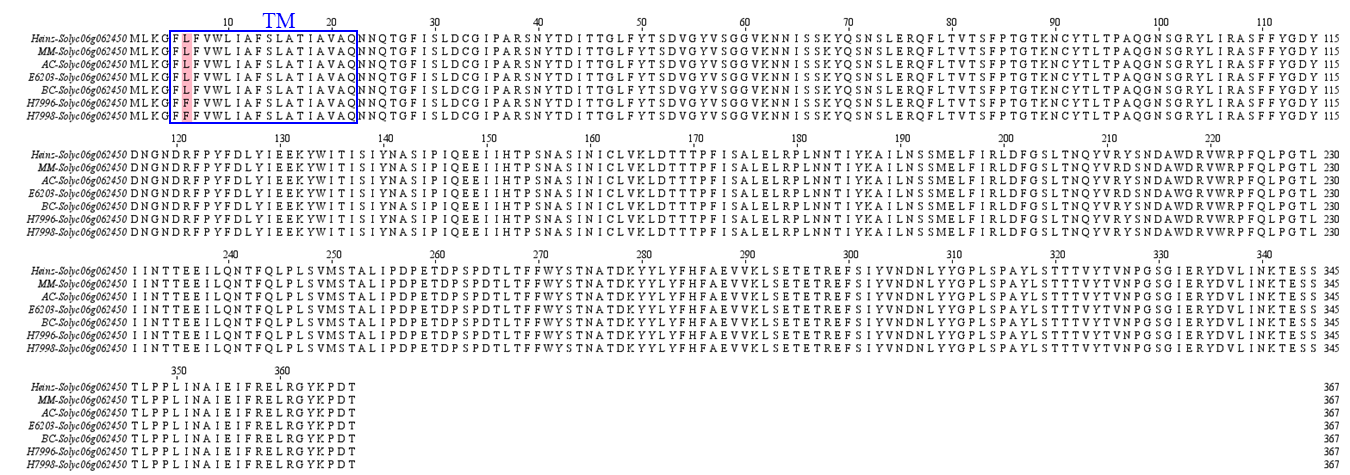


Solyc06g063150.3.1 - Receptor-like kinase (RLK)


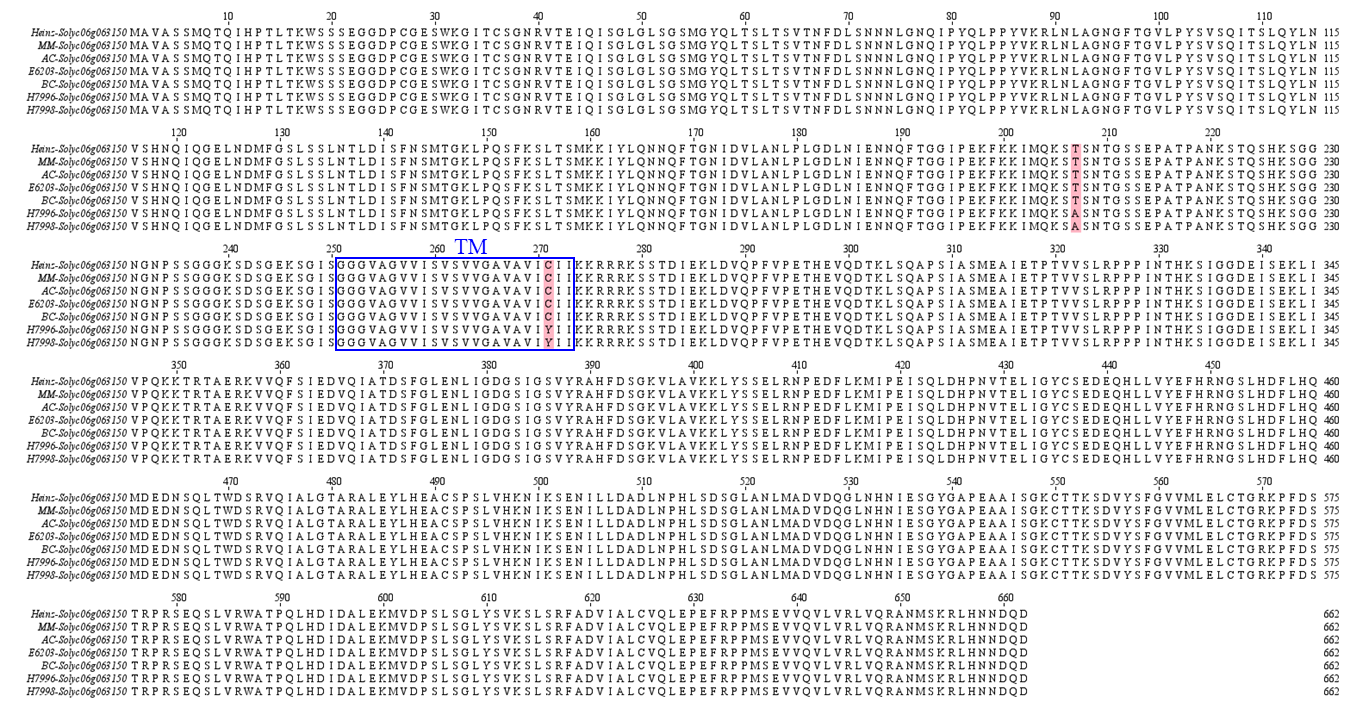


Solyc06g064680.1.1 - Nucleotide-binding domain leucine-rich repeat (NLR)


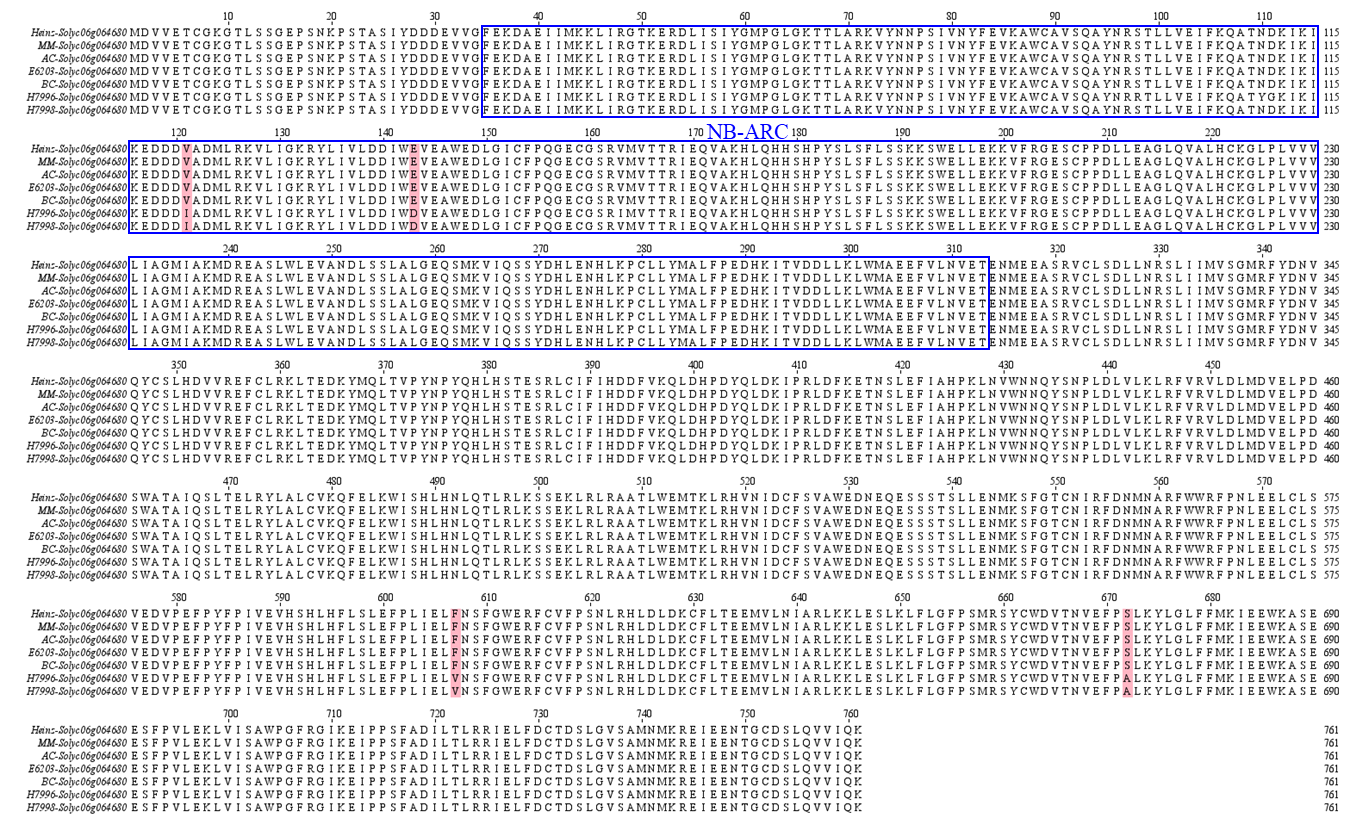


Solyc06g064720.1.1- Nucleotide-binding domain leucine-rich repeat (NLR)


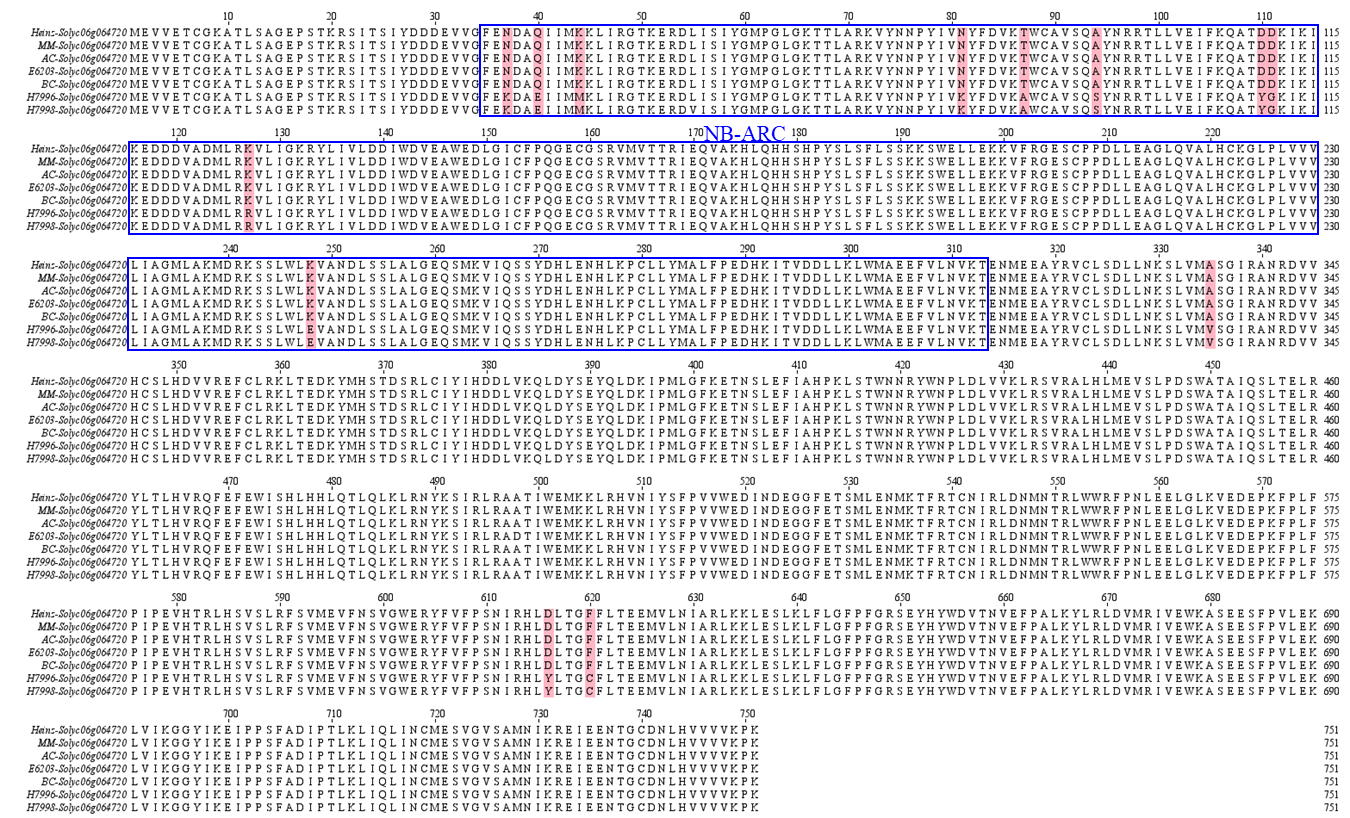


Solyc06g064750.1.1 - Nucleotide-binding domain leucine-rich repeat (NLR)


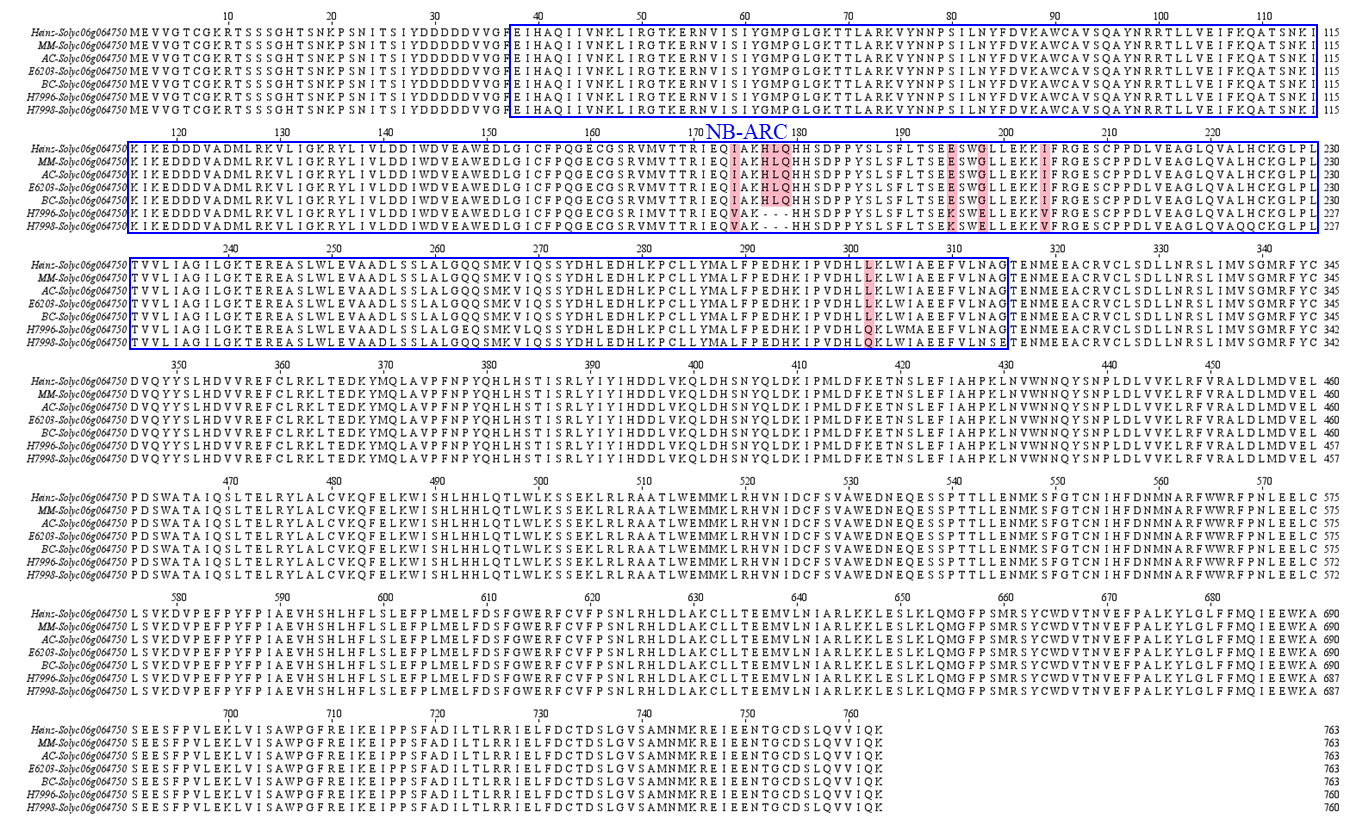


Solyc06g064760.1.1 - Nucleotide-binding domain leucine-rich repeat (NLR)


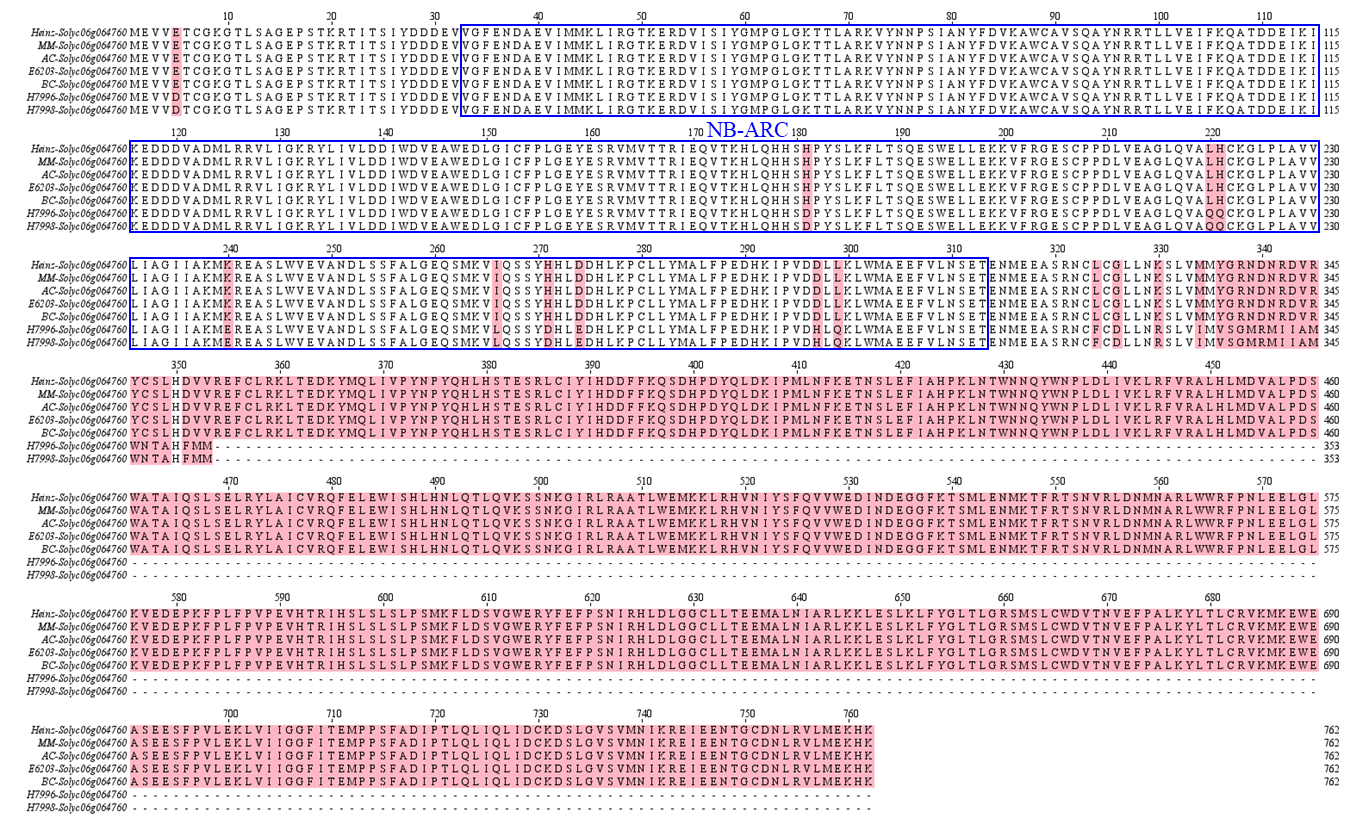


Solyc06g065150.1.1 - Receptor-like protein (RLP)

**
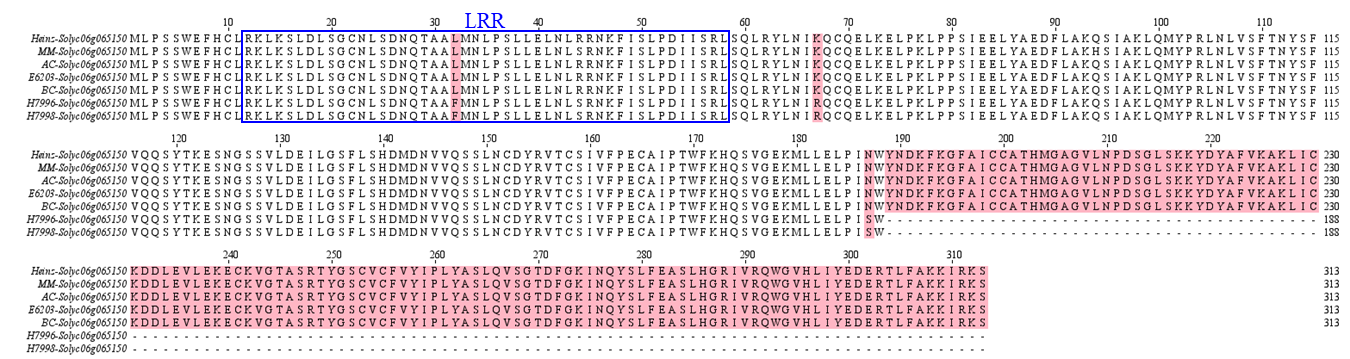
**

**Supplementary Figure 7** Amino acid sequence alignment of nine candidate genes harboring mutations in putative functional domains between five susceptible and two resistant cultivars. Susceptible cultivars are Heinz 1706 (Heinz), Moneymaker (MM), Ailsa Craig (AC), E6203, Black Cherry (BC), and resistant cultivars are Hawaii 7996 (H7996) and Hawaii 7998 (H7998). Polymorphic sequences between the susceptible and resistant lines are highlighted in red. Protein domains harboring putative functional mutations are indicated in blue box. TM, transmembrane; NB-ARC, nucleotide-binding adaptor shared by APAF-1, R proteins, and CED-4; LRR, leucine-rich repeat.


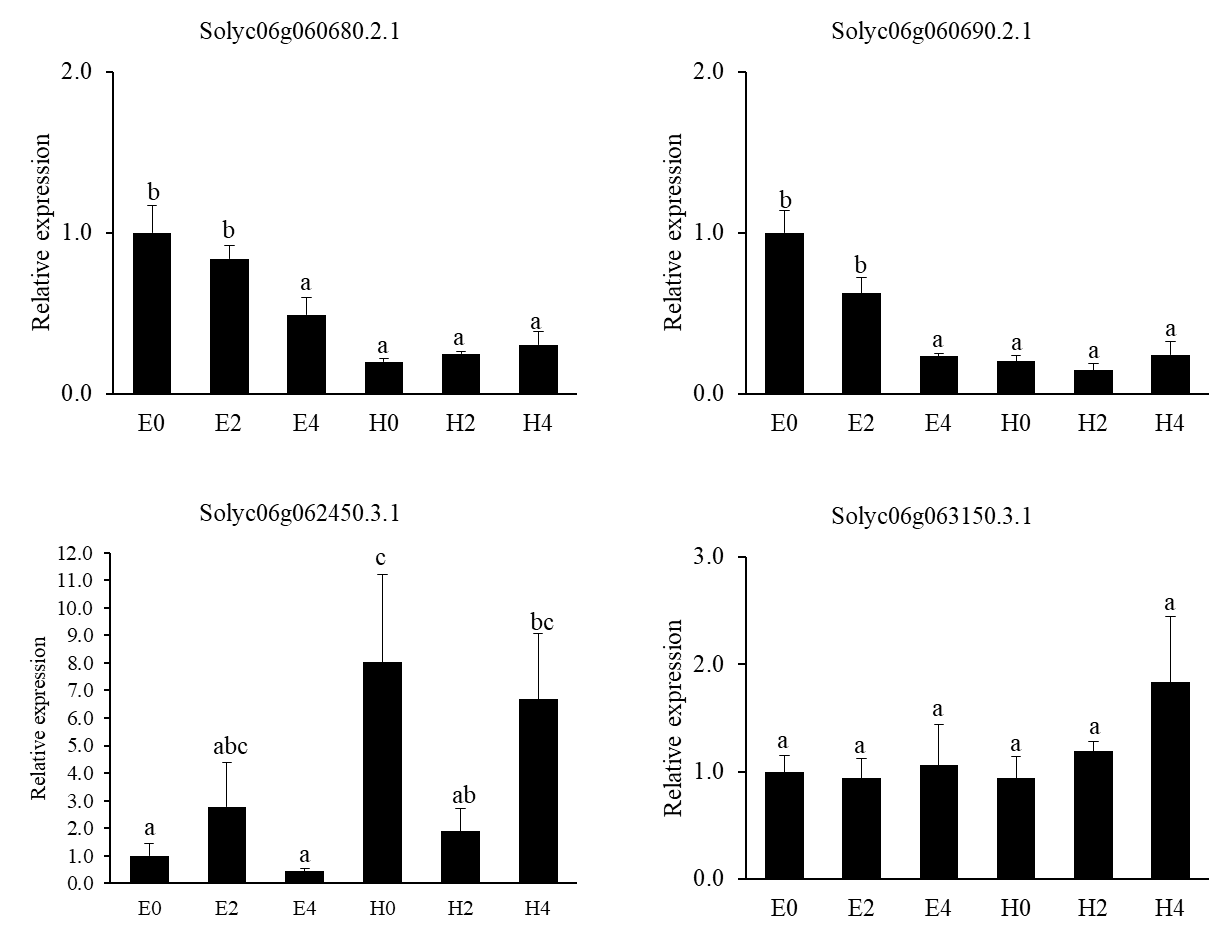


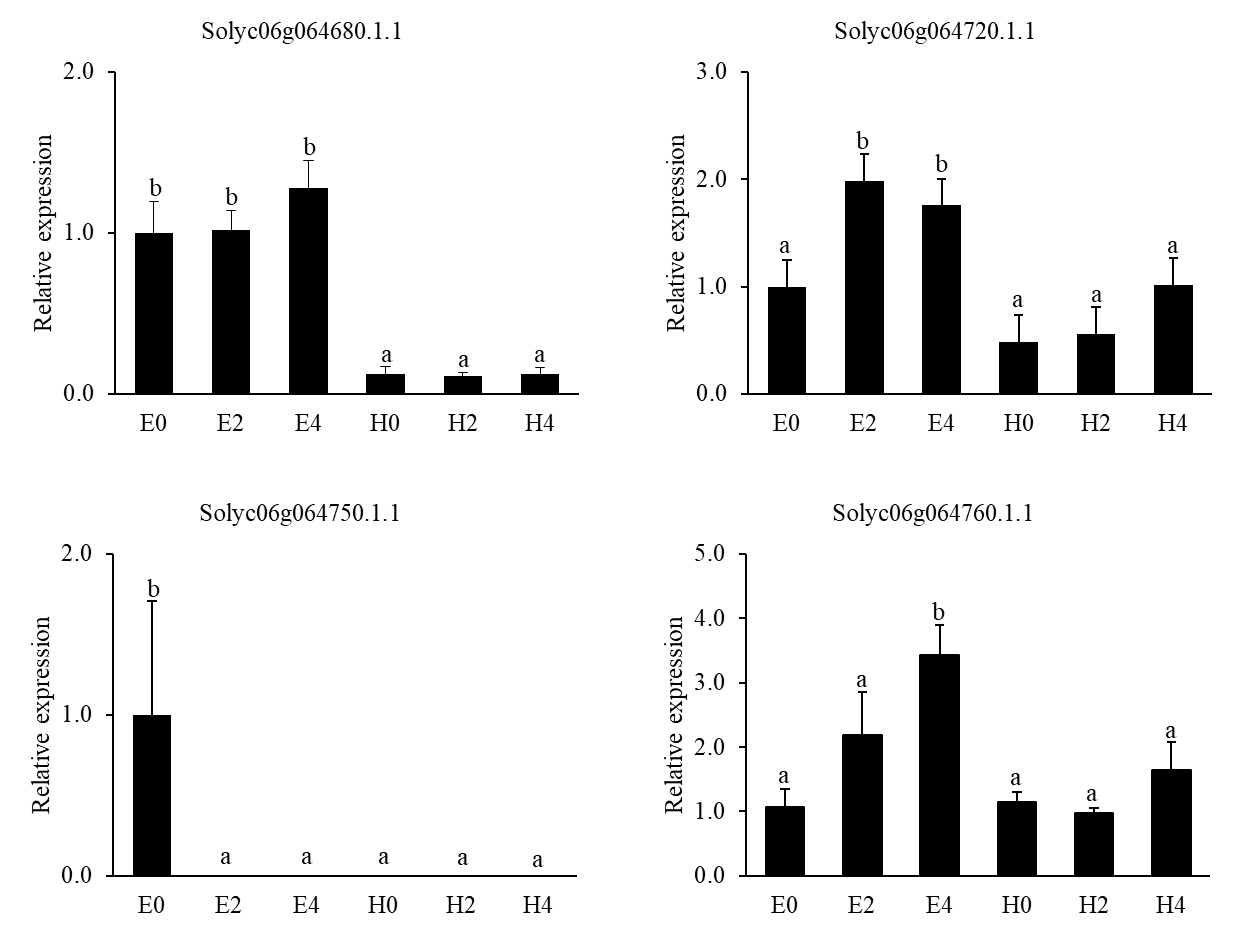


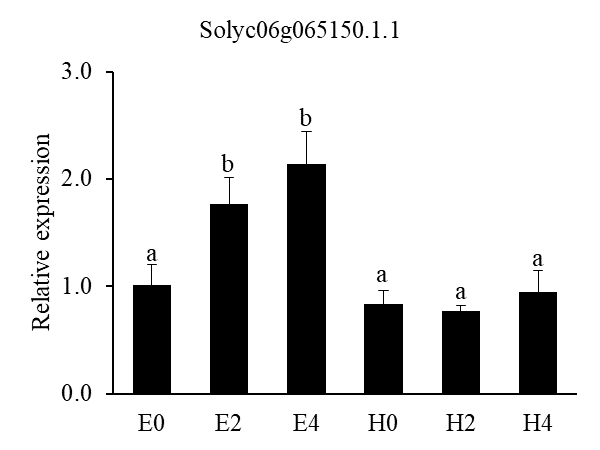


**Supplementary Figure 8** Relative expression levels of candidate genes at 0 days post-inoculation (dpi, mock inoculation), 2 dpi, and 4 dpi in leaf tissues of E6203 (E) and Hawaii 7998 (H) by qRT-PCR. Relative expression was normalized to *GAPDH* (g*lyceraldehyde 3-phosphate dehydrogenase*). The data represent means ± standard error (n=6). Different letters on the bars indicate significant difference (*p* < 0.05) according to Duncan’s multiple range test.
